# Supplementary material for: Activatable probes for diagnosing and positioning liver injury and metastatic tumors by multispectral optoacoustic tomography
Source: Nat Commun. 2018 Sep 28;9:3983. doi: 10.1038/s41467-018-06499-1 (PMC6162313; doi:10.1038/s41467-018-06499-1)
Supplement: Supplementary file 1 — Supplementary Information [file 41467_2018_6499_MOESM1_ESM.pdf]

SUPPLEMENTARY INFORMATION

Activatable probes for diagnosing and positioning liver injury and metastatic tumors by multispectral optoacoustic tomography

Wu et al

Contents

Supplementary Figure 1 – Synthesis route
Supplementary Figures 2-19 – NMR and mass spectra
Supplementary Figure 20 – OA signal intensities versus wavelength
Supplementary Figure 21 – Responses of C^1X-OR^1 to ALP in solution
Supplementary Figure 22 – Responses of C^2X-OR^2 to β-galactosidase in solution
Supplementary Figure 23 – Sensitivity determination for activated probes
Supplementary Figure 24 – Cell viabilities, flow cytometry profiles for cellular uptake and fluorescent microscopic images for C^1X-OR^1
Supplementary Figure 25 – Cell viabilities, flow cytometry profiles for cellular uptake and fluorescent microscopic images for C^2X-OR^2
Supplementary Figure 26 – Blood biochemical assays, body weight and histological analysis for C^1X-OR^1
Supplementary Figure 27 – Blood biochemical assays, body weight and histological analysis for C^2X-OR^2
Supplementary Figure 28 – Cross-sectional MSOT images of a mouse at varied time points upon injection of C^1X-OR^1
Supplementary Figure 29 – MSOT images at three different cross sections of APAP-treated mouse
Supplementary Figure 30 – Cross-sectional and z-stack MIP MSOT images for a mouse at 0, 6 and 18 h post injection of 300 mg kg^{-1} of APAP
Supplementary Figure 31 – MSOT imaging, H&E staining of liver section and serum ALP levels
Supplementary Figure 32 – Detected MSOT spectra
Supplementary Figure 33 – MSOT images for a mouse at 0, 2, 4, and 8 h after combined treatment of TNF-α/D-GAL
Supplementary Figure 34 – Western blotting analyses of activated caspase-3 and H&E staining for TNF-α/D-GAL treated mice
Supplementary Figure 35 – Western blotting analyses for different liver lobes
Supplementary Figure 36 – Serum ALP level for the mice upon N-acetylcysteine treatment
Supplementary Figure 37 – MSOT and fluorescence images for tumor-bearing mice
Supplementary Figure 38 – Cross-sectional images for lymphatic metastasis
Supplementary Figure 39 – Immunohistochemical analysis of CD206
Supplementary Methods
Supplementary References

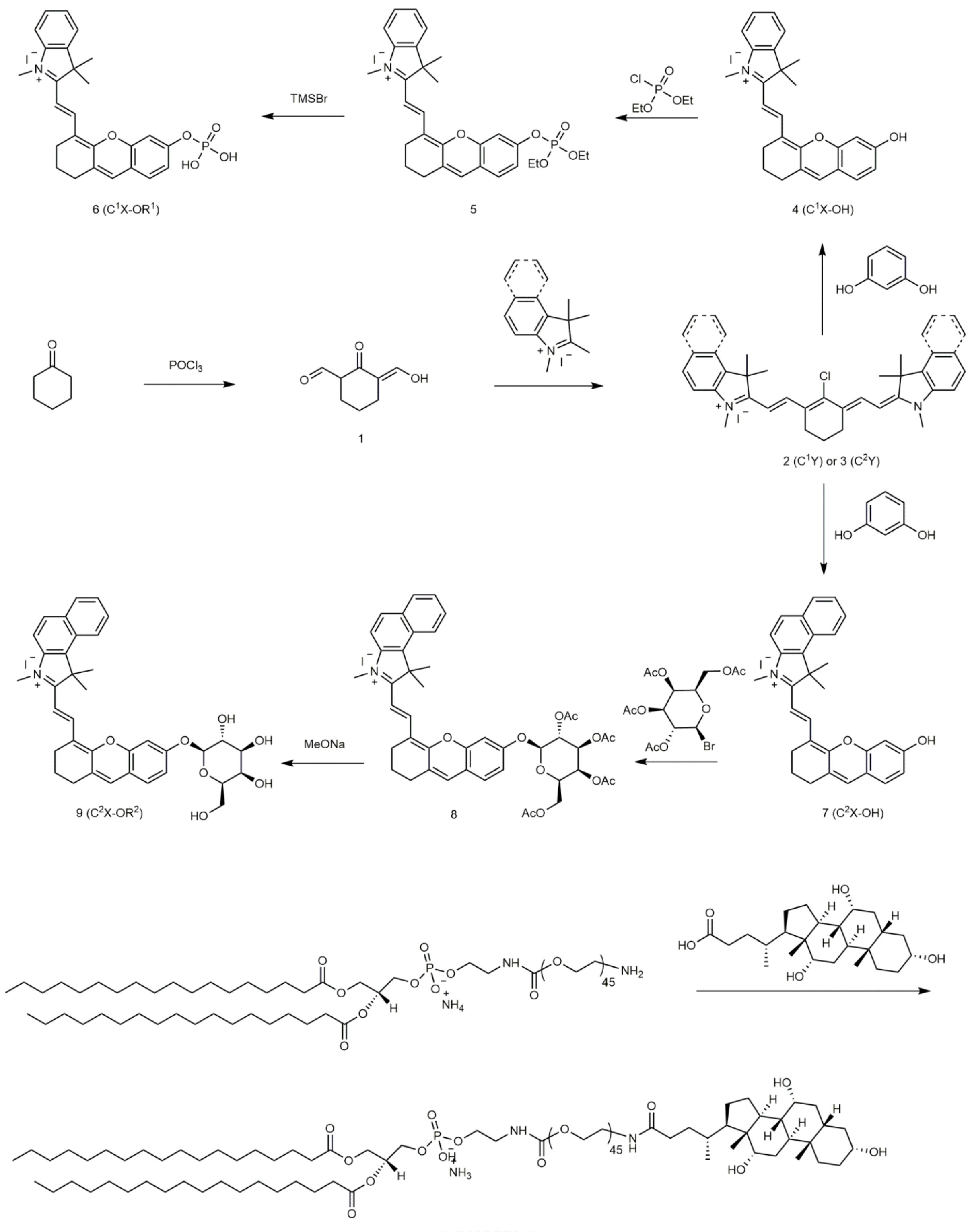

Supplementary Figure 1. Synthesis route of the two molecular MSOT probes as well as liver-targeting phospholipid.

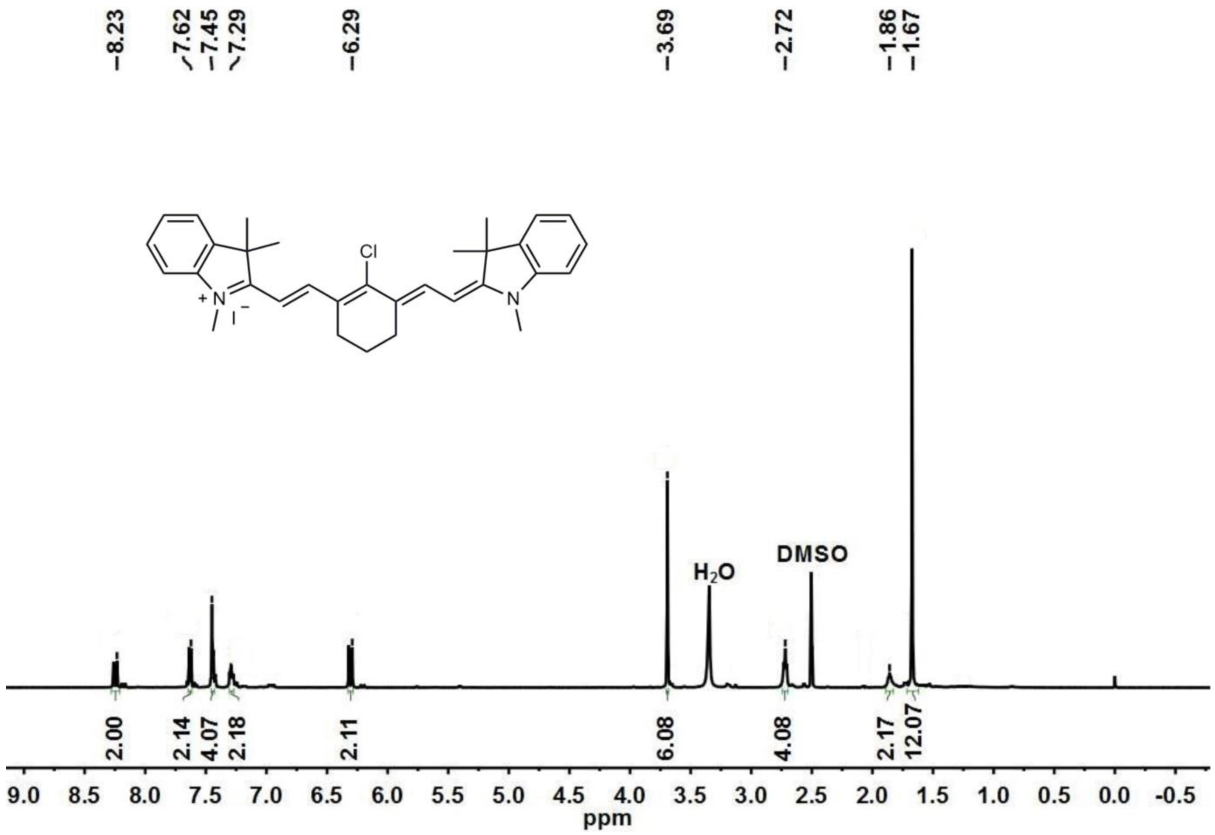

Supplementary Figure 2. ^{1}H NMR spectrum of $C^{1}Y$ (DMSO-d_6, 600 MHz).

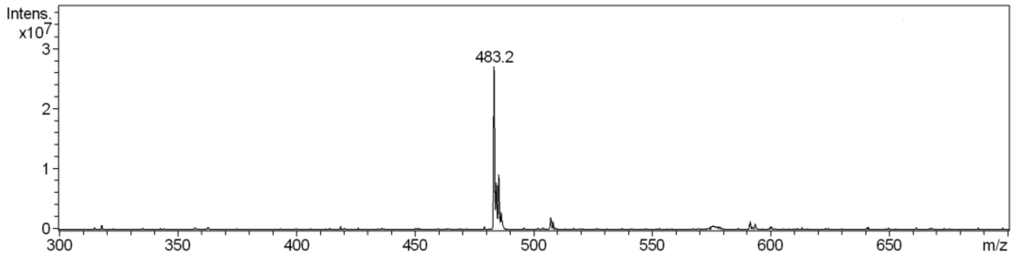

Supplementary Figure 3. Mass spectrum of $C^{1}Y$. MS (ESI): *m/z* 483.2 $[M]^{+}$.

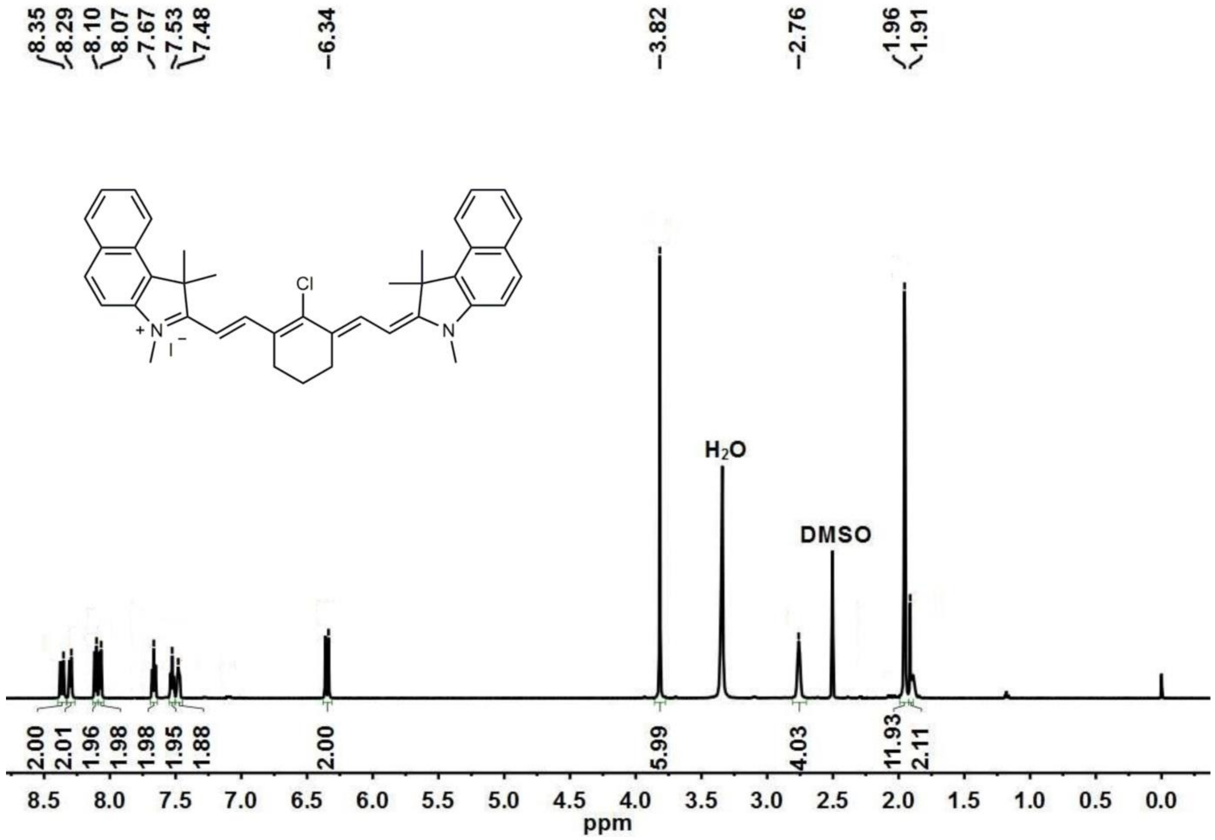

Supplementary Figure 4. ^{1}H NMR spectrum of C^2Y (DMSO-d_6, 600 MHz).

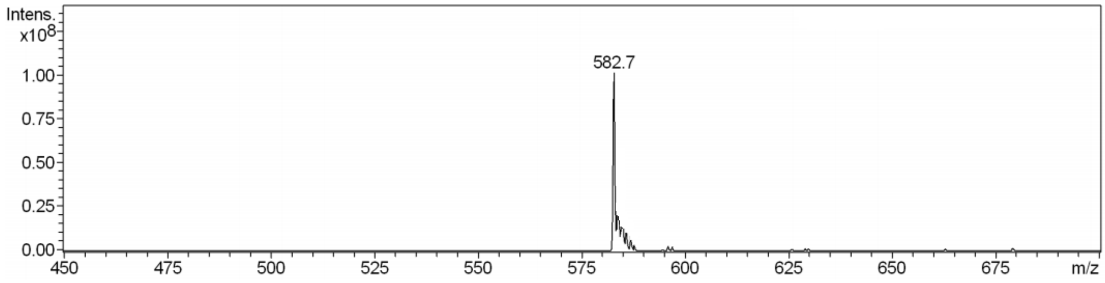

Supplementary Figure 5. Mass spectrum of C^2Y. MS (ESI): *m/z* 582.7 $[M]^+$.

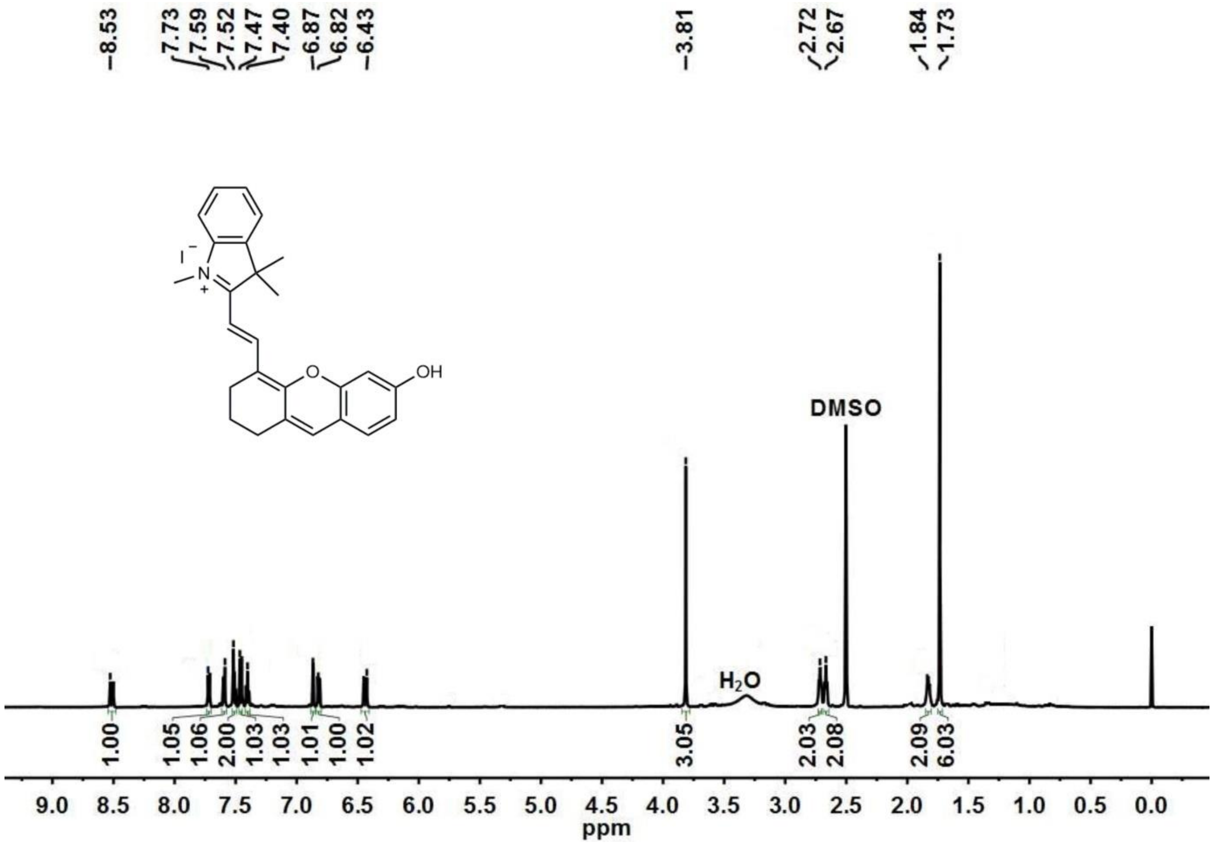

Supplementary Figure 6. ^{1}H NMR spectrum of $C^{1}X$-OH (DMSO-d_6, 600 MHz).

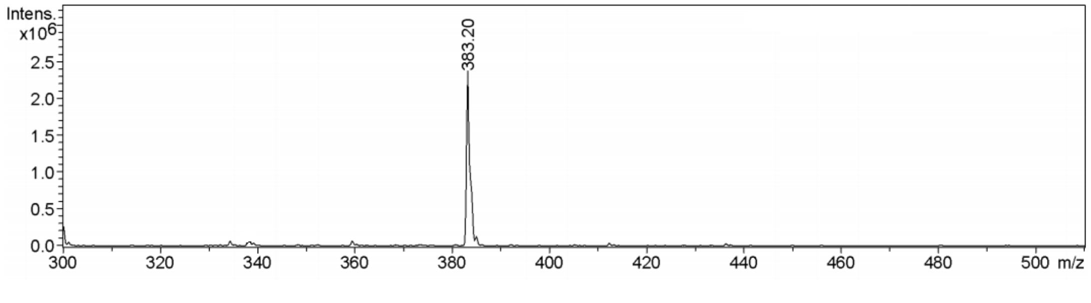

Supplementary Figure 7. Mass spectrum of $C^{1}X$-OH. MS (ESI): *m/z* 383.20 $[M]^{+}$.

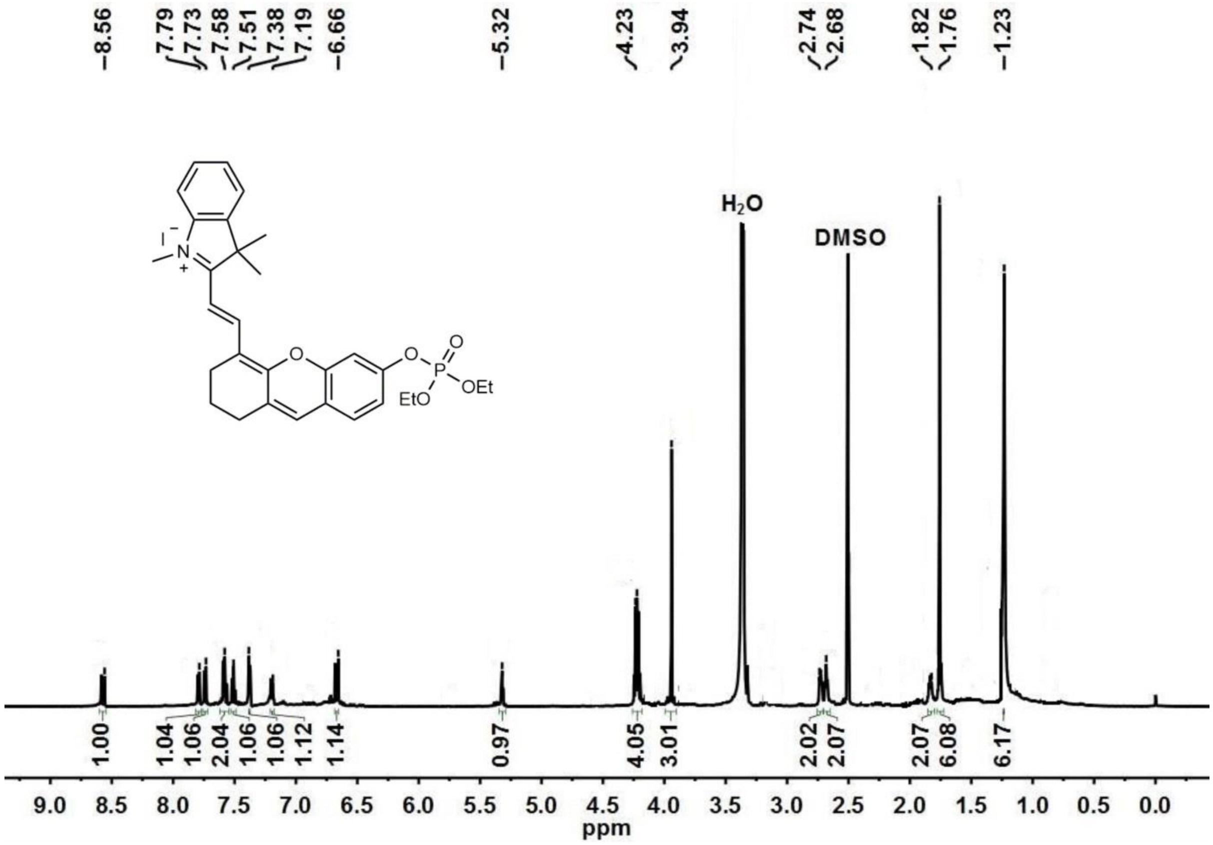

Supplementary Figure 8. ^{1}H NMR spectrum of compound 5 (DMSO-d_6, 600 MHz).

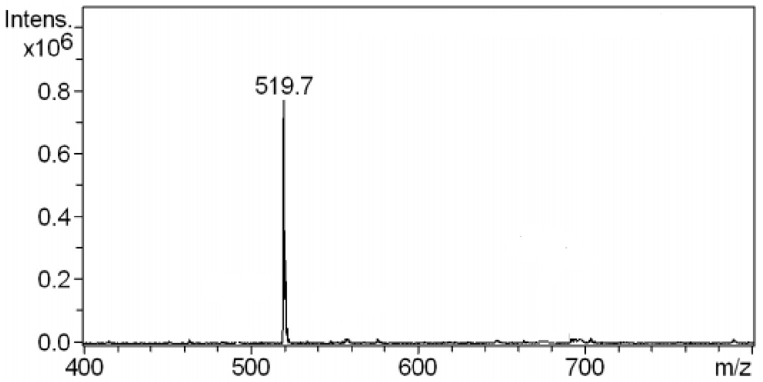

Supplementary Figure 9. Mass spectrum of compound 5. MS (ESI): *m/z* 519.7 $[M]^{+}$.

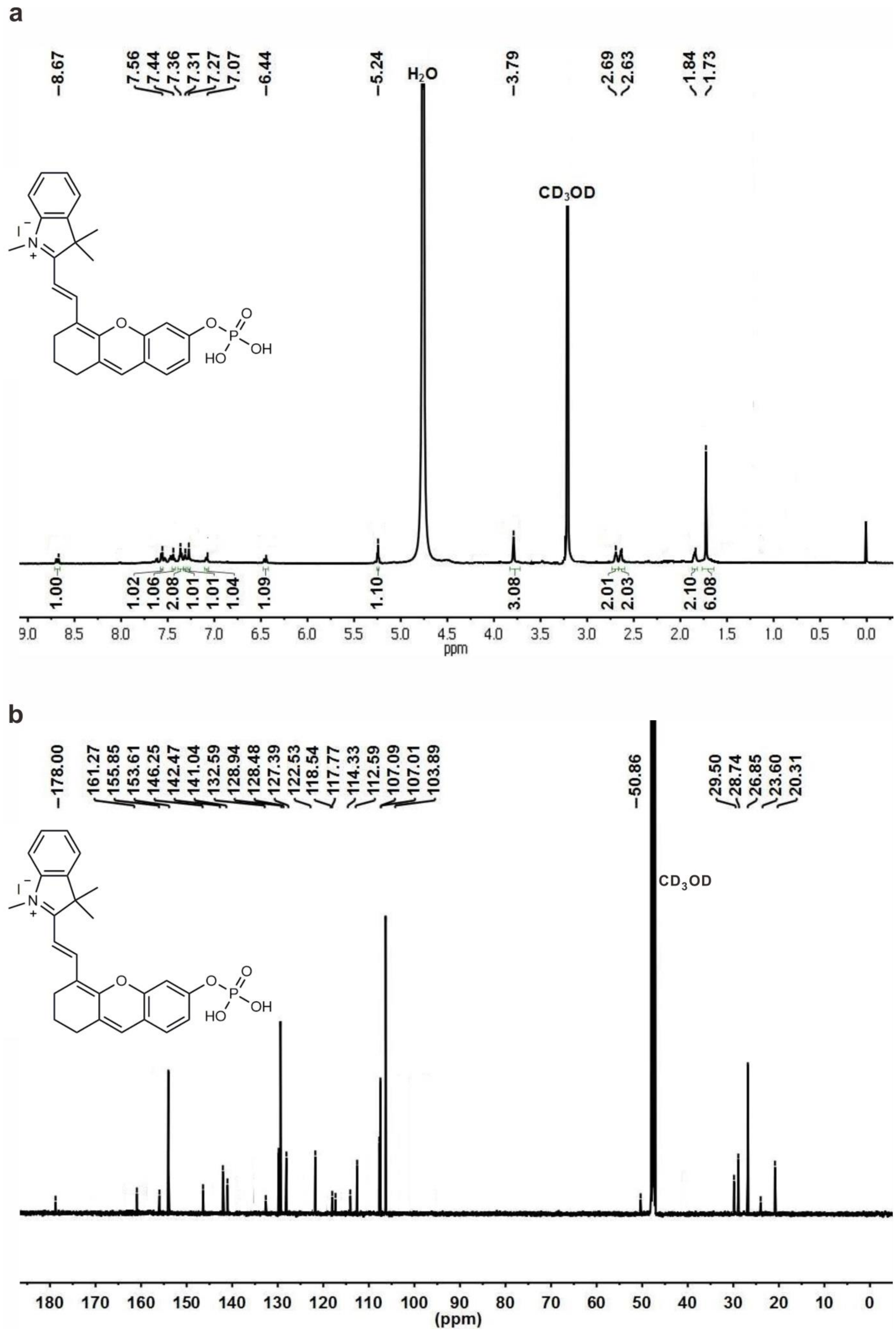

Supplementary Figure 10. ^{1}H and ^{13}C NMR spectra of $C^{1}X\text{-}OR^{1}$ (CD_3OD, 600 MHz).

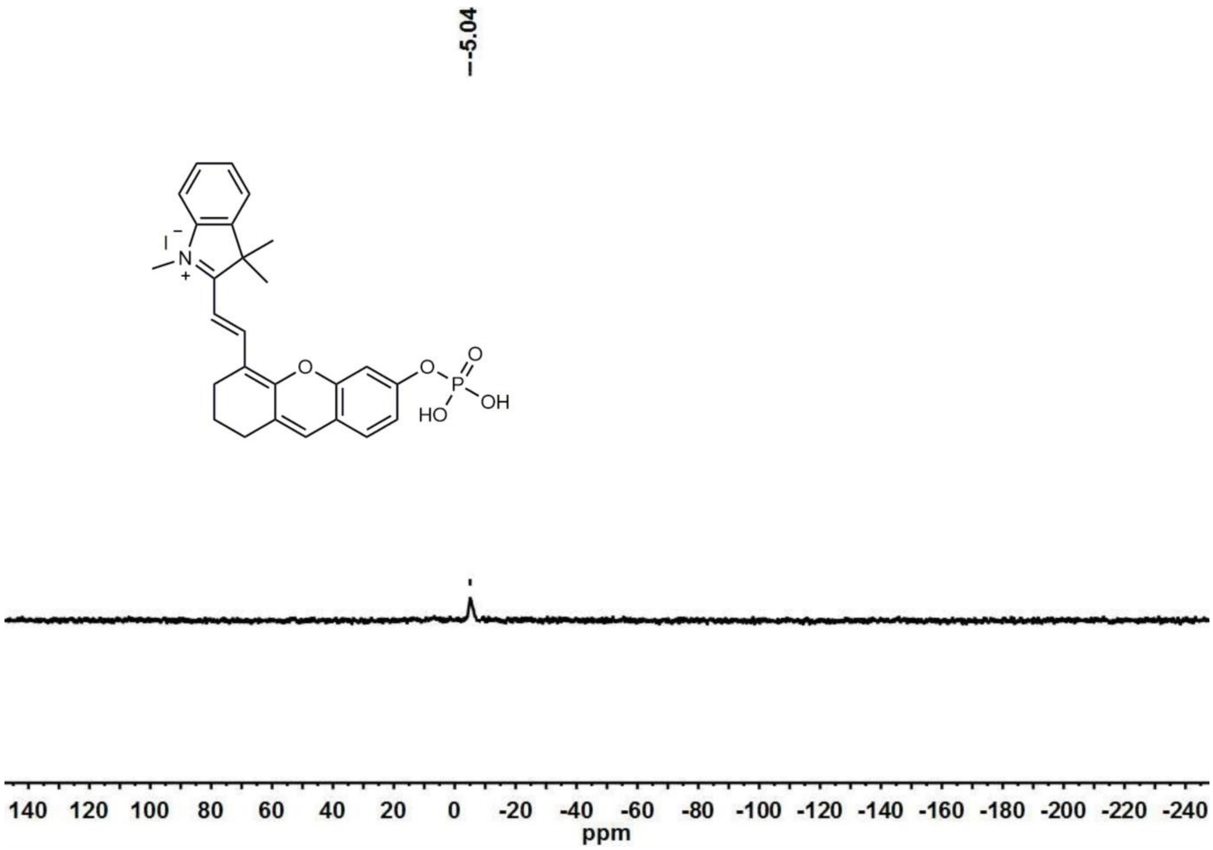

Supplementary Figure 11. ^{31}P NMR spectrum of C[1]X-OR[1] (CD_3OD, 600 MHz).

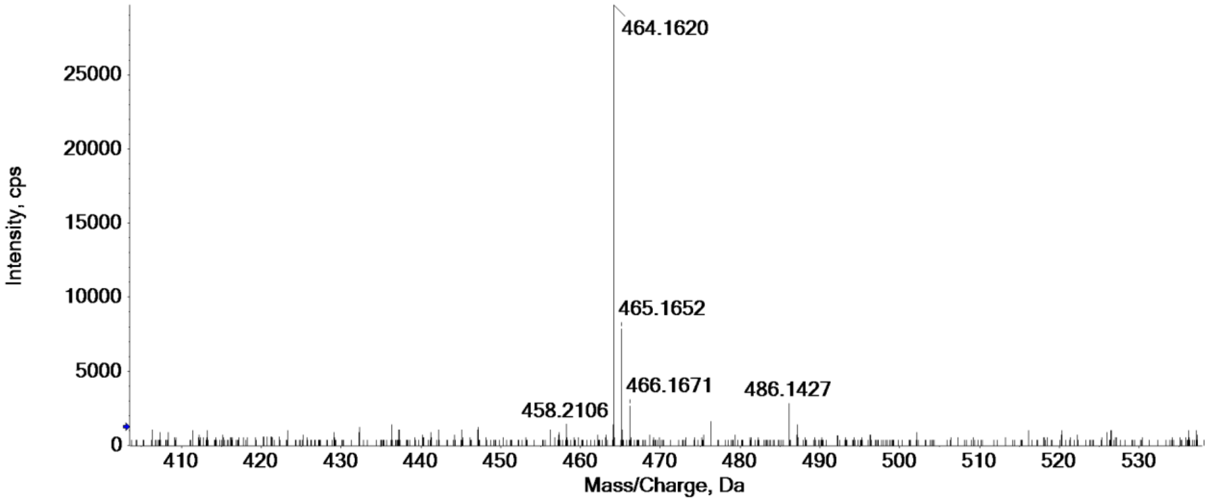

Supplementary Figure 12. High resolution mass spectrum of C^1X-OR^1. *m/z* 464.1620 $[M]^+$.

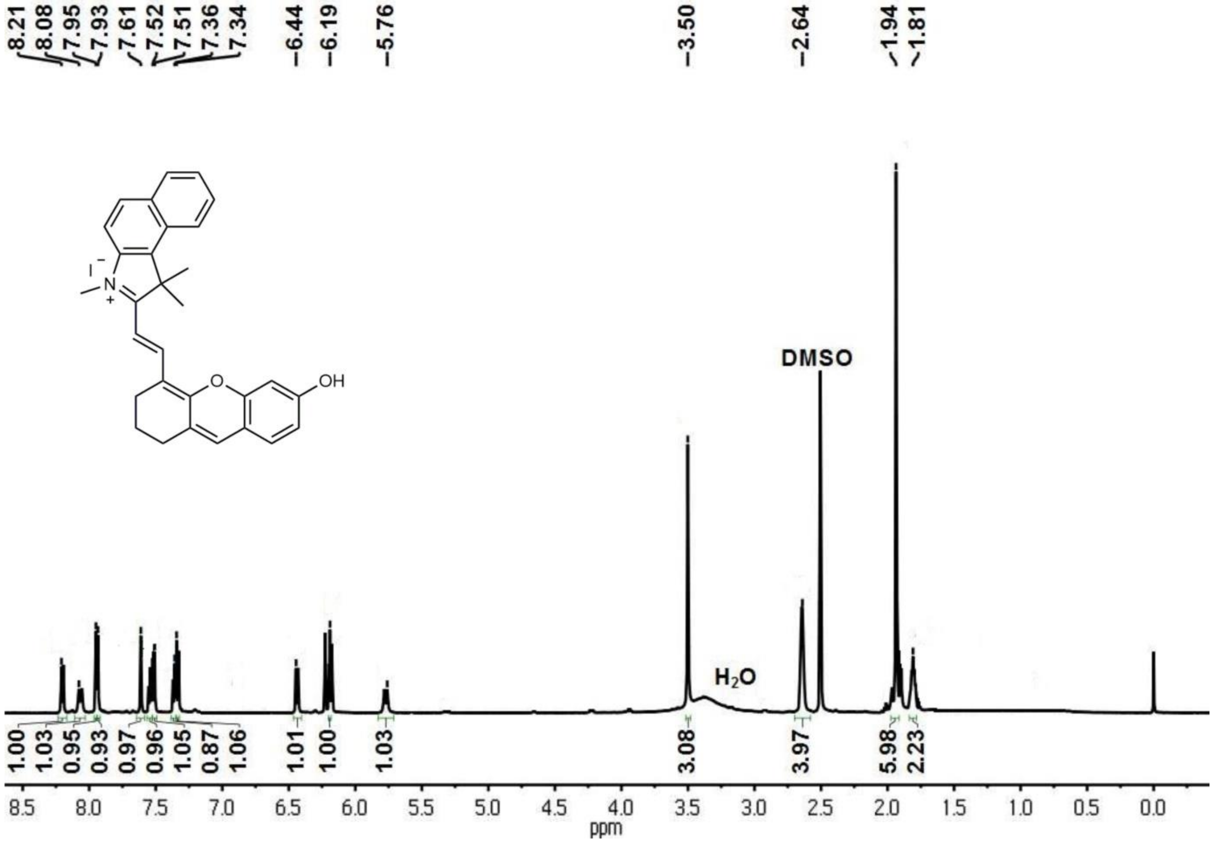

Supplementary Figure 13. ^1H NMR spectrum of C^2X-OH (DMSO-d_6, 600 MHz).

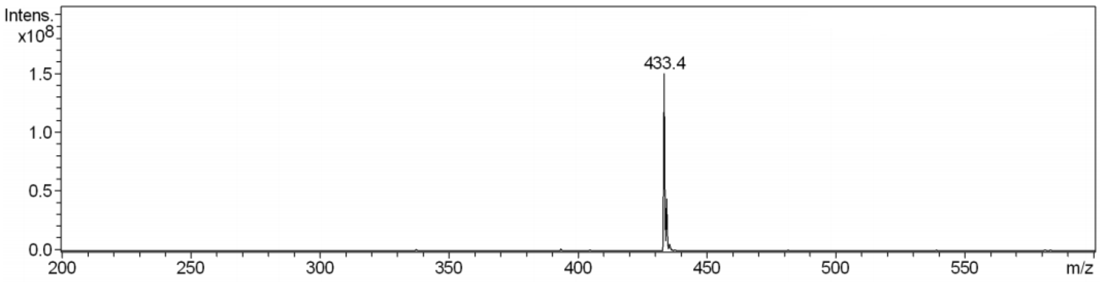

Supplementary Figure 14. Mass spectrum of C^2X-OH. MS (ESI): *m/z* 433.4 $[M]^+$.

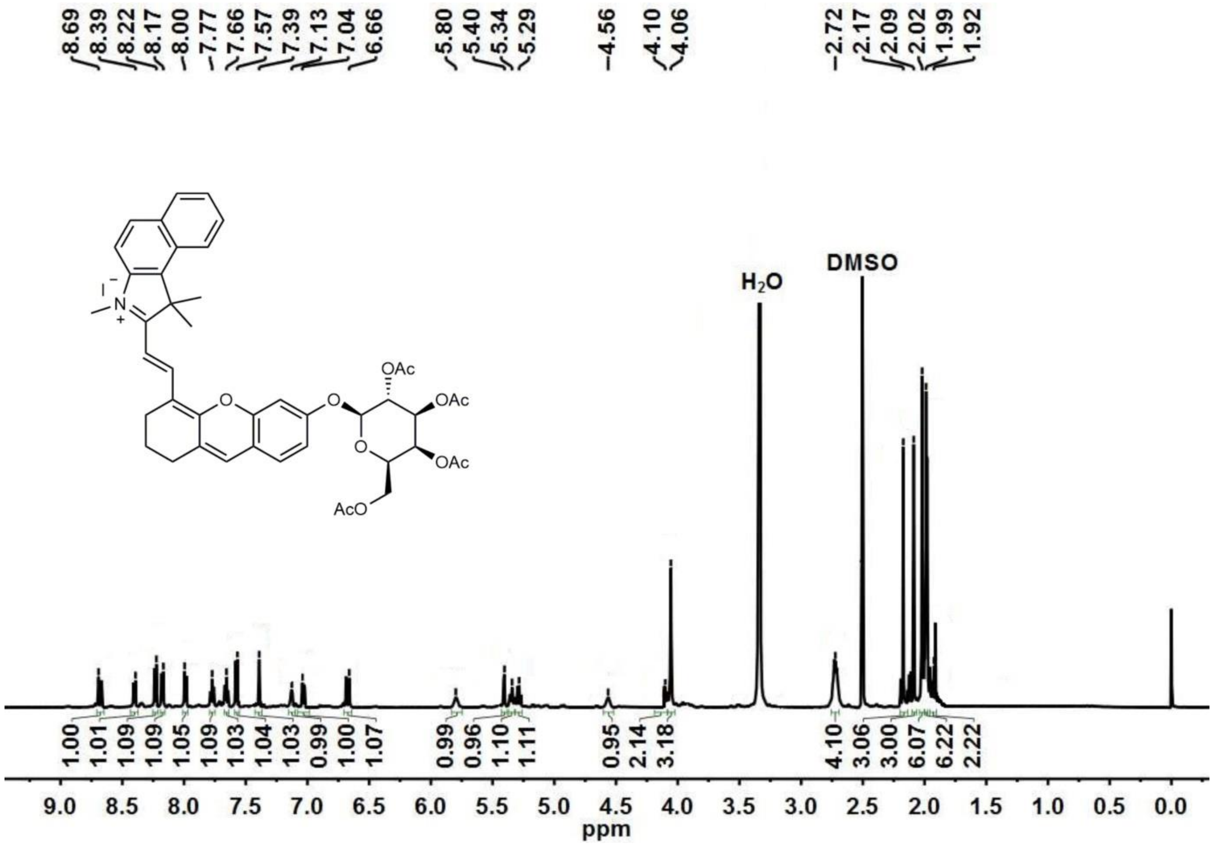

Supplementary Figure 15. ^1H NMR spectrum of compound 8 (DMSO-d_6, 600 MHz).

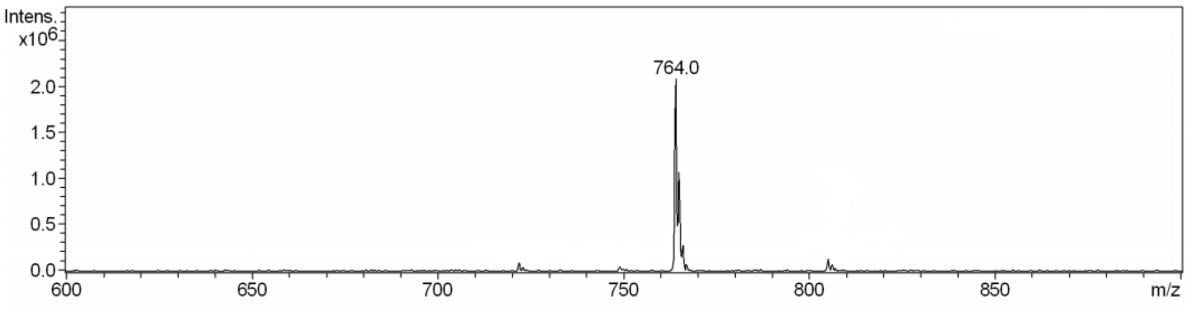

Supplementary Figure 16. Mass spectrum of compound 8 MS (ESI): *m/z* 764.0 $[M]^+$.

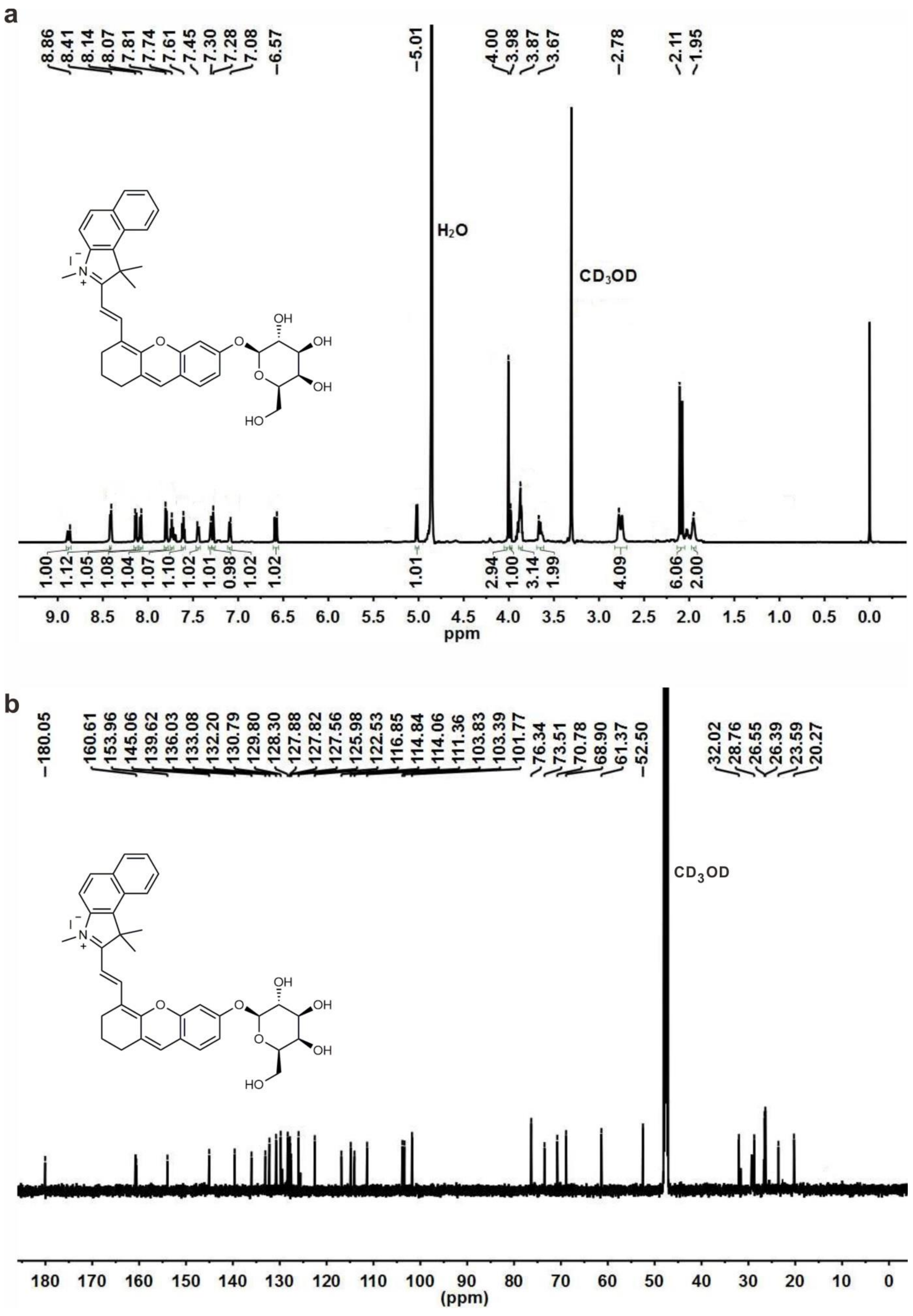

Supplementary Figure 17. ^{1}H and ^{13}C NMR spectra of C^2X-OR^2 (CD_3OD, 600 MHz).

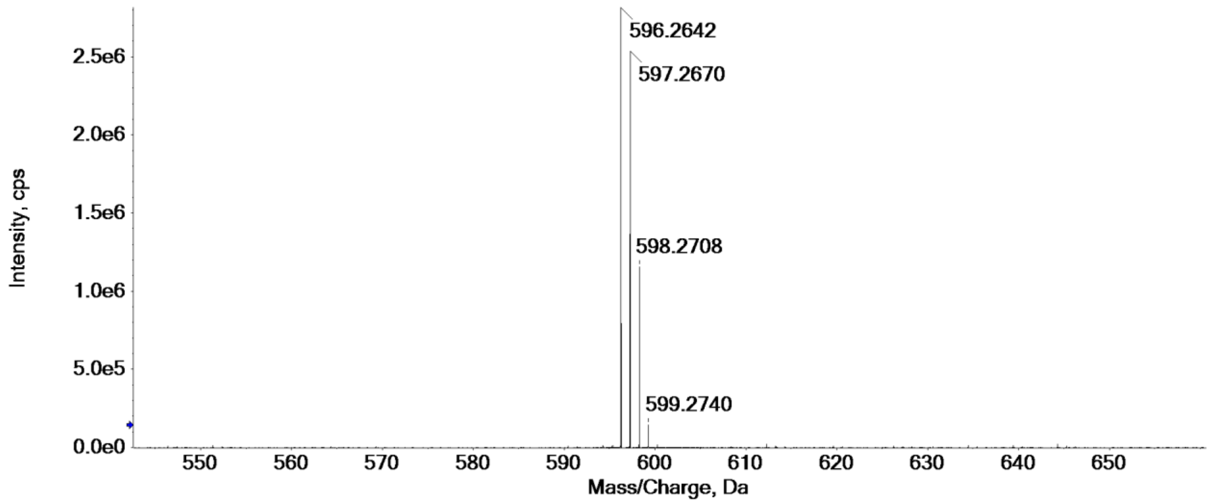

Supplementary Figure 18. High resolution mass spectrum (HR-MS) of C^{2}X-OR^2. *m/z* 596.2642 $[M]^+$

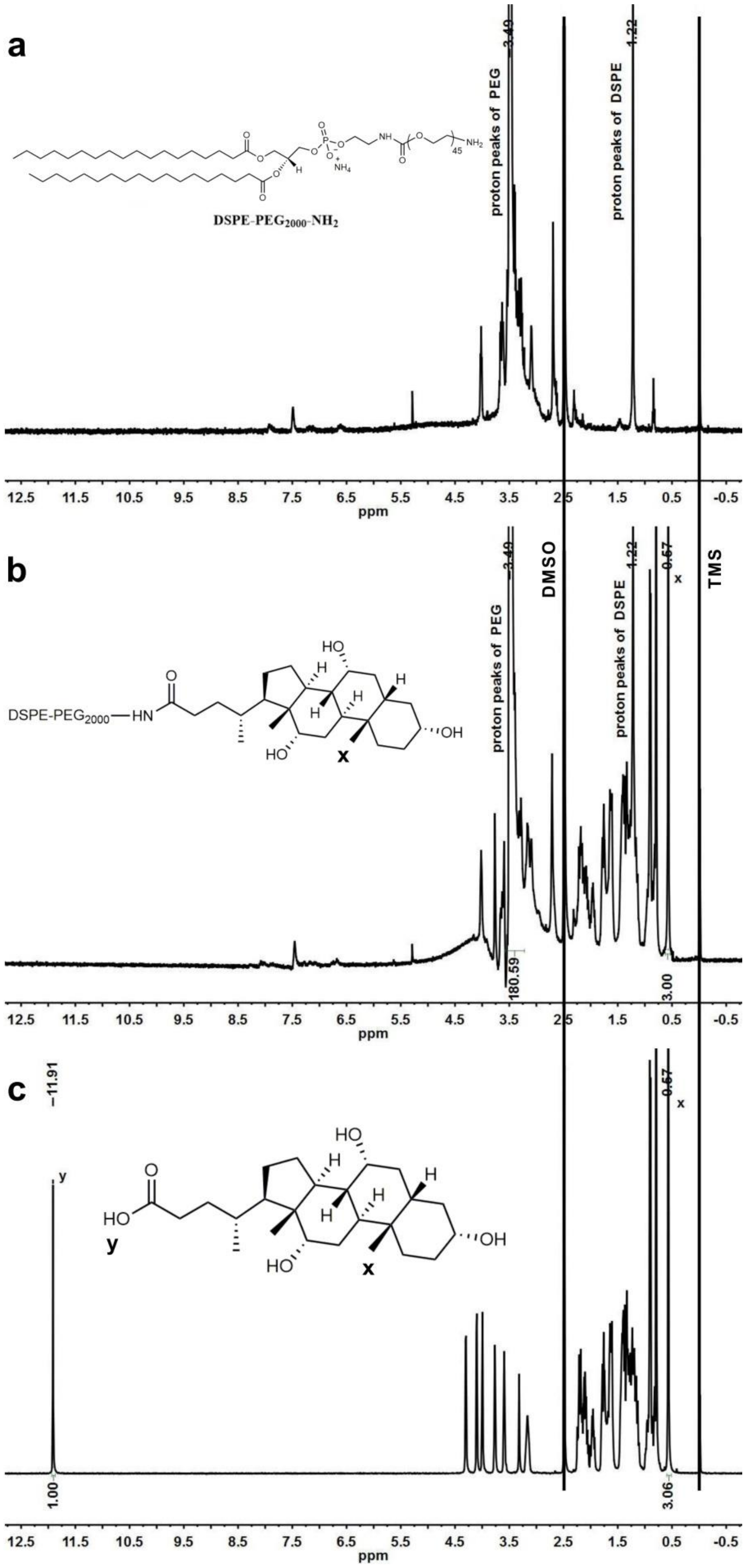

Supplementary Figure 19. ^{1}H NMR spectra. (**a**) DSPE-PEG$_{2000}$-NH$_2$, (**b**) DSPE-PEG$_{2000}$-ChA, (**c**) cholic acid (ChA) (DMSO-d$_6$, 600 MHz).

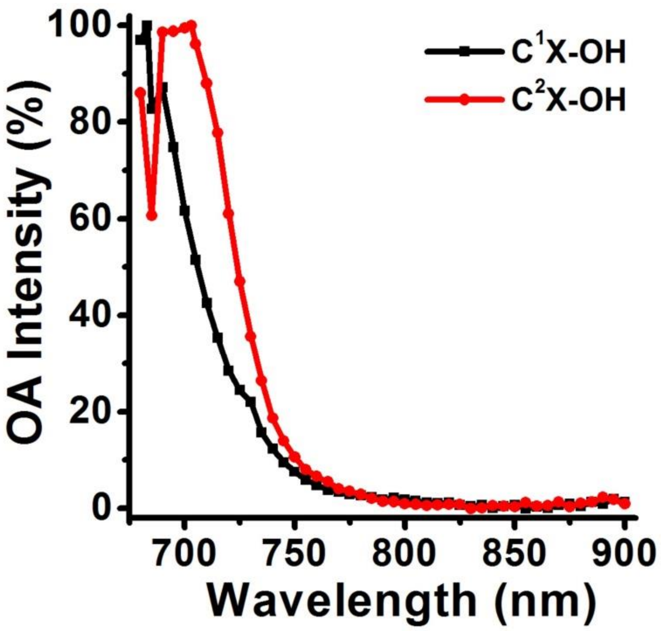

Supplementary Figure 20. Normalized OA signal intensities as a function of wavelength for C^1X-OH in TRIS solution (pH = 8.0) and C^2X-OH in PBS solution (pH = 7.4).

(OA signal intensity of C^1X-OH and C^2X-OH in phantom was recorded with different excitation wavelengths respectively).

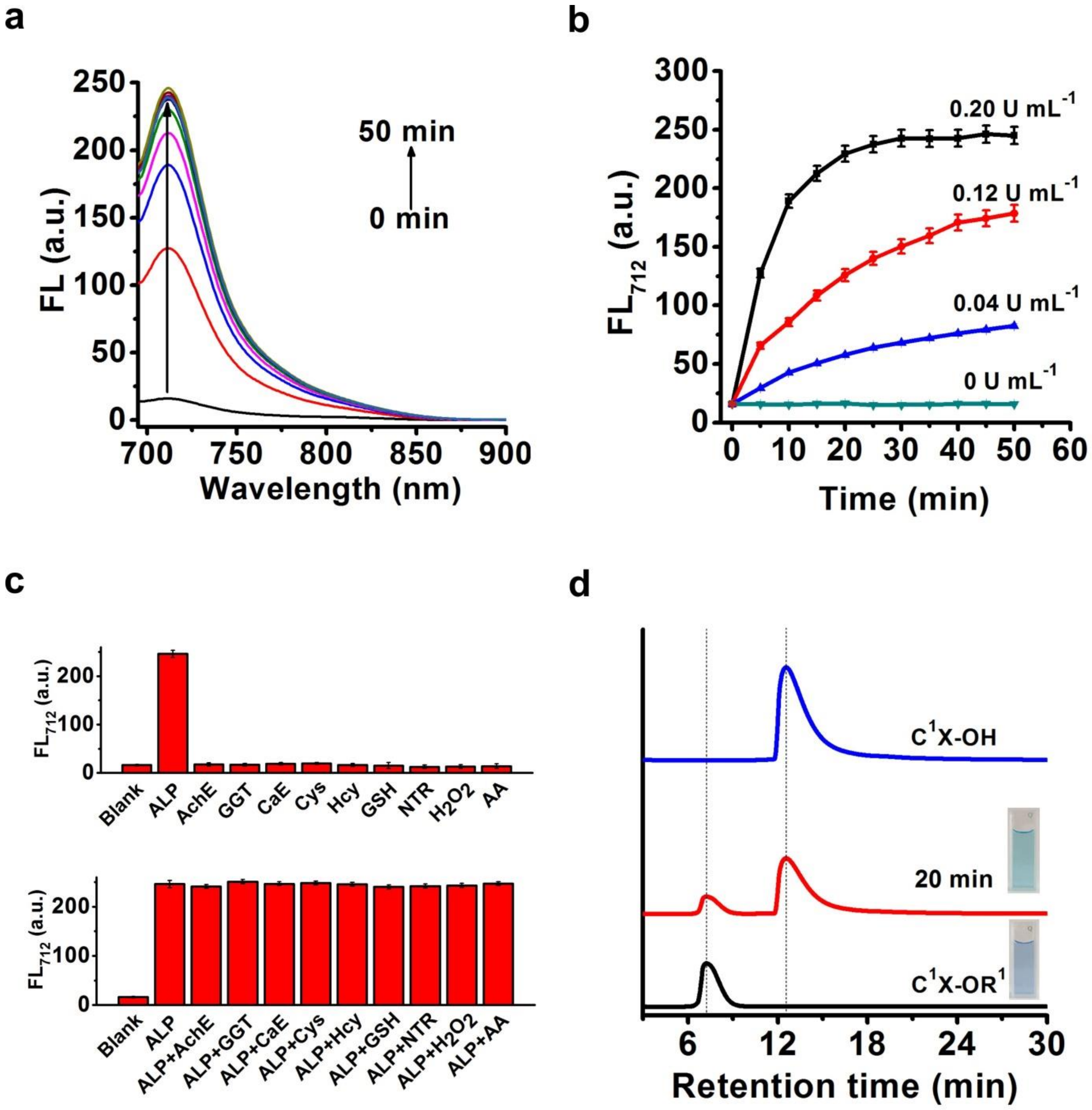

Supplementary Figure 21. Responses of C^1X-OR^1 to ALP in solution. (**a**) Time-dependent emission spectra of C^1X-OR^1 in the presence of ALP 0.20 U mL^{-1}; (**b**) fluorescence intensity at 712 nm as a function of time in the presence of different ALP levels (n = 3); (**c**) upper panel: fluorescence intensity of C^1X-OR^1 (5 μM) in the presence of 0.20 U mL^{-1} ALP or the same amount of other enzymes respectively. lower panel: fluorescence emission intensity of C^1X-OR^1 (5 μM) in the presence of 0.20 U mL^{-1} ALP and another substance, respectively. All fluorescence intensities

were measured 30 min after the addition of substances (excitation wavelength: 680 nm) (n = 3). (**d**) HPLC data of pure $C^1X\text{-}OR^1$, mixture of $C^1X\text{-}OR^1$ and ALP upon reaction for 20 min and pure $C^1X\text{-}OH$. Mobile phase: methanol/acetonitrile/water = 75/15/10. Error bars represent the standard deviation (SD). Data were represented as mean ± SD.

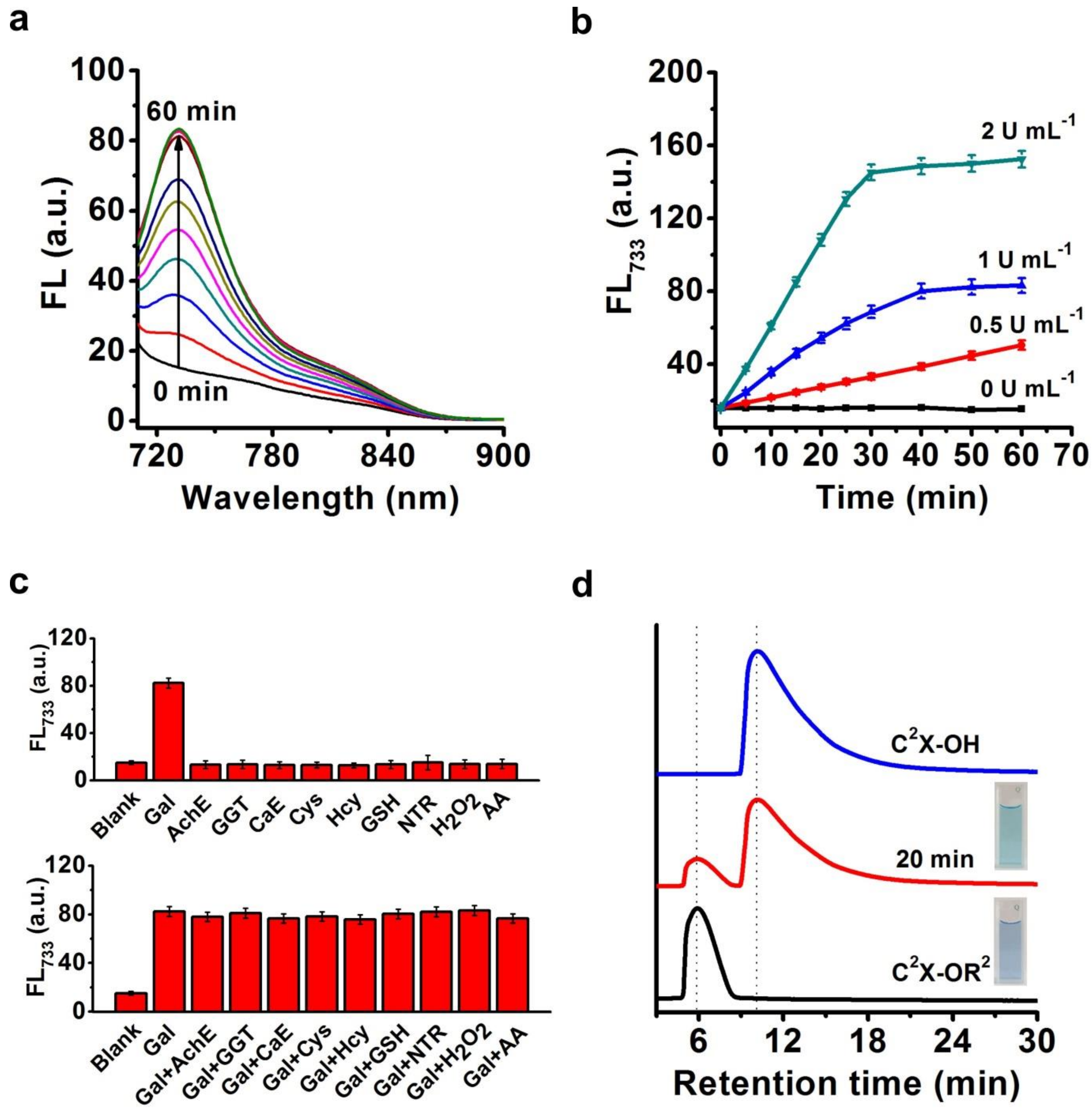

Supplementary Figure 22. Responses of $C^2X\text{-}OR^2$ to β-galactosidase in solution. (**a**) Time-dependent emission spectra of $C^2X\text{-}OR^2$ in the presence of 1 U mL^{-1} β-galactosidase; (**b**)

fluorescence intensity at 733 nm as a function of time in the presence of different ALP levels (n = 3); (**c**) upper panel: fluorescence intensity of C^2X-OR^2 (5 μM) in the presence of 1 U mL^{-1} β-galactosidase or the same amount of other substances respectively. Lower panel: fluorescence emission intensity of C^2X-OR^2 (5 μM) in the presence of 1 U mL^{-1} β-galactosidase and another substance, respectively. All fluorescence intensities were measured 30 min after the addition of enzymes (excitation wavelength: 680 nm) (n = 3). (**d**) HPLC data of pure C^2X-OR^2, mixture of C^2X-OR^2 and β-galactosidase upon reaction for 20 min and pure C^2X-OH. Mobile phase: methanol/ water = 85/15. Error bars represent the standard deviation (SD). Data were represented as mean ± SD.

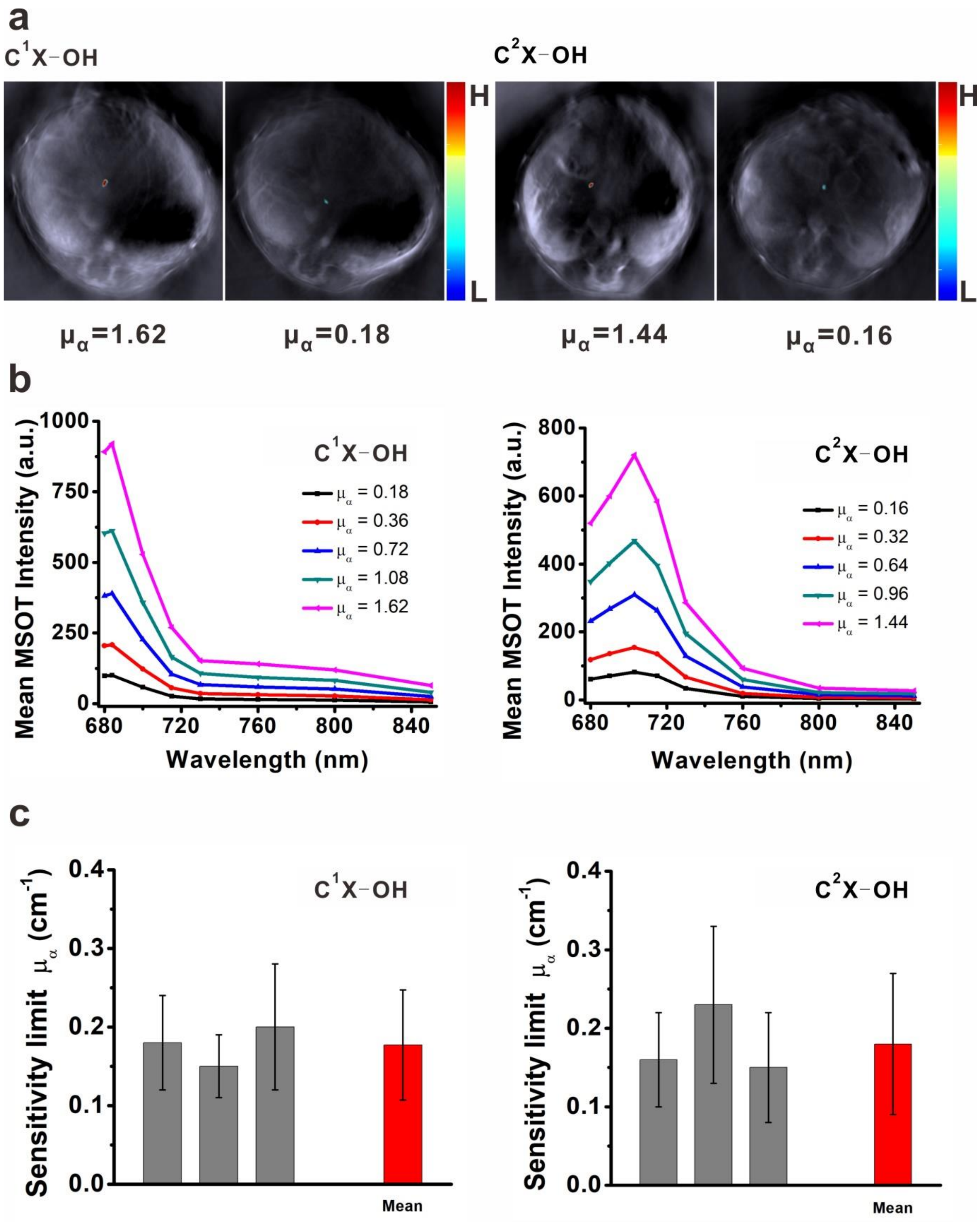

Supplementary Figure 23. Determination of sensitivity of the two activated probes (C^1X-OH and C^2X-OH) in euthanized mice. (a) Image examples of detection results using two activated probes with two peak absorption coefficients (1.62 cm^{-1} and 0.18 cm^{-1} for C^1X-OH; 1.44 cm^{-1} and 0.16 cm^{-1} for C^2X-OH). The peak absorption coefficient $\mu_\alpha = \varepsilon \cdot c$ (where ε is the molar extinction coefficient at maximum absorption and *c* is the concentration). (b) Typical curves reflecting the impact of C^1X-OH or C^2X-OH on the spectral background for different concentrations

corresponding to the probes' peak absorption coefficients. The MSOT signals were collected in the region of interests. (c) Determined sensitivity limits of C^1X-OH and C^2X-OH. The bar height corresponds to the mean lowest C^1X-OH (or C^2X-OH) μ_α and the error bar to the standard deviation over all images employed for evaluation. The three grey bars correspond to three different mice and the red bar corresponds to statistics stemming from all data.

The determined sensitivity limits (the criterion for the sensitivity limit determination follows the literature reports [1,2]) for the activated probes are listed in the following table:

Activated probe	μ_α (cm^{-1})	*c* (μM)	ε at maximum absorption ($M^{-1}cm^{-1}$)
C^1X-OH	0.177	2.3	7.7×10^4
C^2X-OH	0.180	3.3	5.4×10^4

Note: with the sensitivity limits of 2.3 μM and 3.3 μM, the two activated probe are sensitive enough to detect the disease biomarkers. The injected dosages were 3.7 mg kg^{-1} (i.v. injection of C^1X-OR^1), 6.4 mg kg^{-1} (intraperitoneal injection of C^2X-OR^2) and 0.64 mg kg^{-1} (footpad tumor injection of C^2X-OR^2) respectively. Given that the two probes are targeted probes and can accumulate in the liver (lysosomal C^1X-OR^1 has liver targeting groups on its surface) or the ovarian tumor (the β-galactose moiety in C^2X-OR^2 can target the β-D-galactose receptors overexpressed on ovarian tumor cells) [3], and upon activation the probes can be readily detected by MSOT facility and thus can function well in diagnosis of corresponding disease.

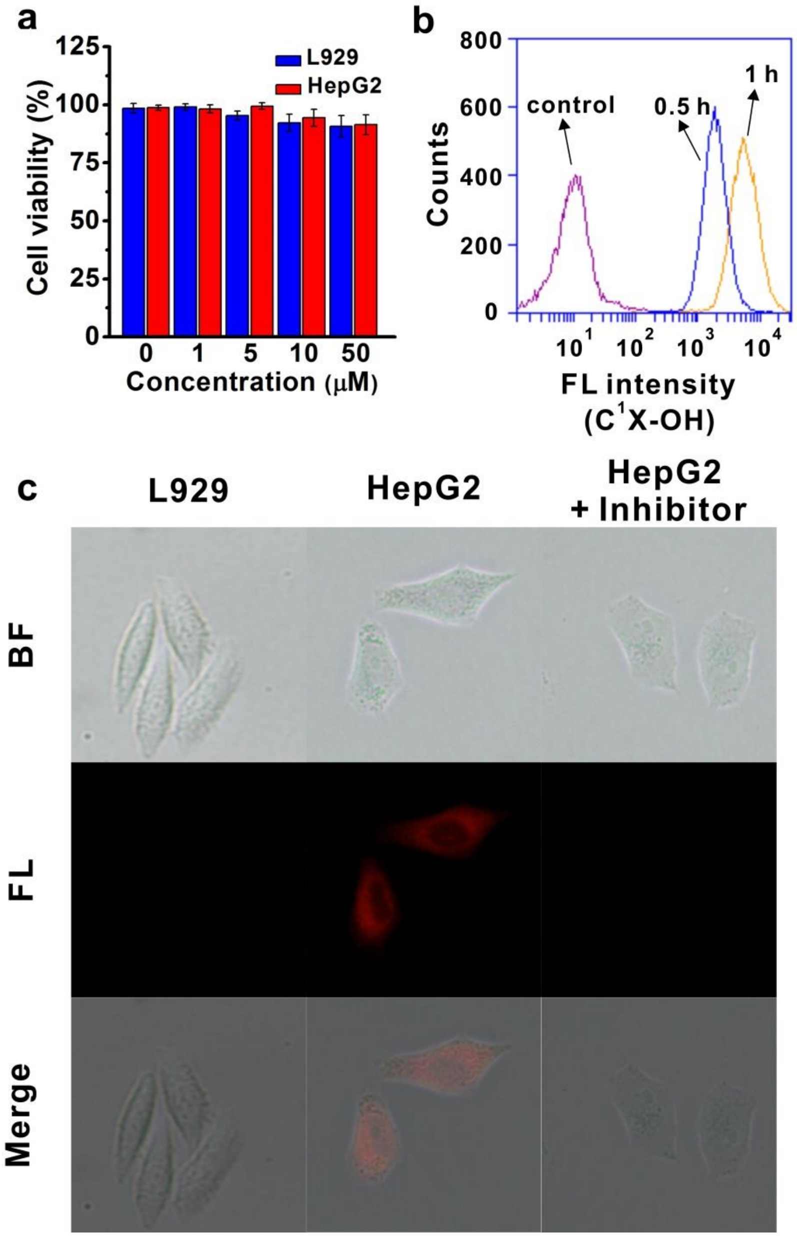

Supplementary Figure 24. Cell viabilities, flow cytometry profiles for cellular uptake and fluorescent microscopic images. (**a**) Viabilities of L929 and HepG2 cells upon 24 hours of incubation with $C^1X\text{-}OR^1$ of different concentrations (0, 1, 5, 10 and 50 μM). Three independent experiments were performed; and for each independent experiment, the assays were performed in eight replicates. Data were represented as mean ± SD from three independent experiments. Error bars represent the standard deviation (SD). (**b**) Flow cytometry profiles for HepG2 cells in the absence (the control) and presence of $C^1X\text{-}OR^1$ for 0.5 or 1 h. (1×10^4 cells). (**c**) Bright field (top row) and fluorescence images (middle row) for L929 and HepG2 cells incubated with $C^1X\text{-}OR^1$ for 1 h, and images for HepG2 cells pretreated with Na_3VO_4 (an ALP inhibitor) and then incubated with $C^1X\text{-}OR^1$ for 1 h.

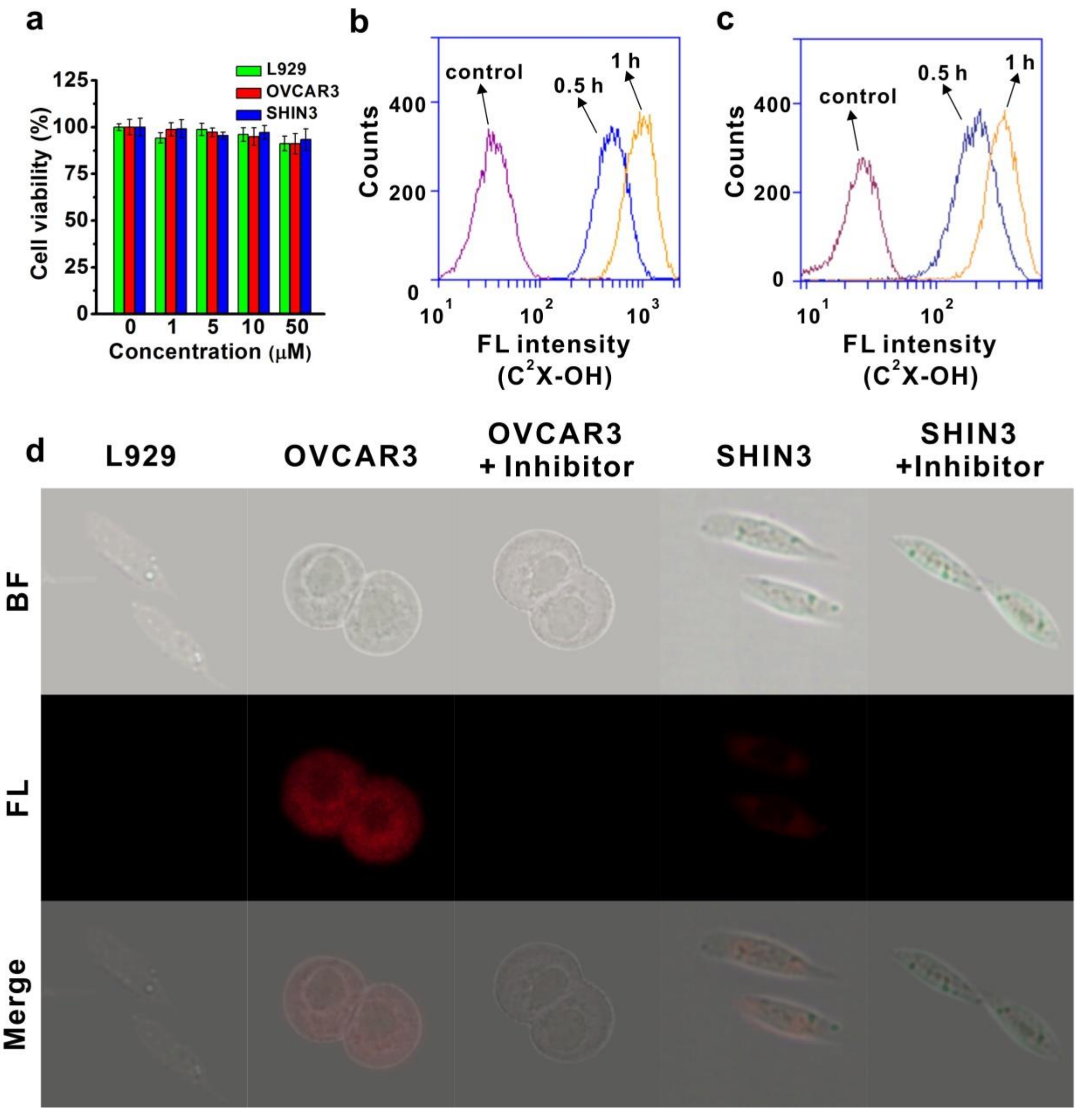

Supplementary Figure 25. Cell viabilities, flow cytometry profiles for cellular uptake and fluorescence images. (**a**) Viabilities of L929, OVCAR3 and SHIN3 cells upon 24 hours incubation with $C^2X\text{-}OR^2$ of different concentrations (0, 1, 5, 10 and 50 μM). Three independent experiments were performed; and for each independent experiment, the assays were performed in eight replicates. Data were represented as mean ± SD from three independent experiments. Columns represent means ± SD. Error bars represent the standard deviation (SD). Flow cytometry profiles for OVCAR3 (**b**) and SHIIN3 cells (**c**) in the absence (the control) and presence of $C^2X\text{-}OR^2$ for 0.5 or 1 h. (1×10^4 cells). (**d**) Bright field (top row) and fluorescence images (middle row) of L929, OVCAR3 and SHIN3 cells incubated with $C^2X\text{-}OR^2$ for 1 h, and the images for OVCAR3 and SHIN3 cells pretreated with D-galactose (a β-galactosidase inhibitor) and then incubated with $C^2X\text{-}OR^2$ for 1 h.

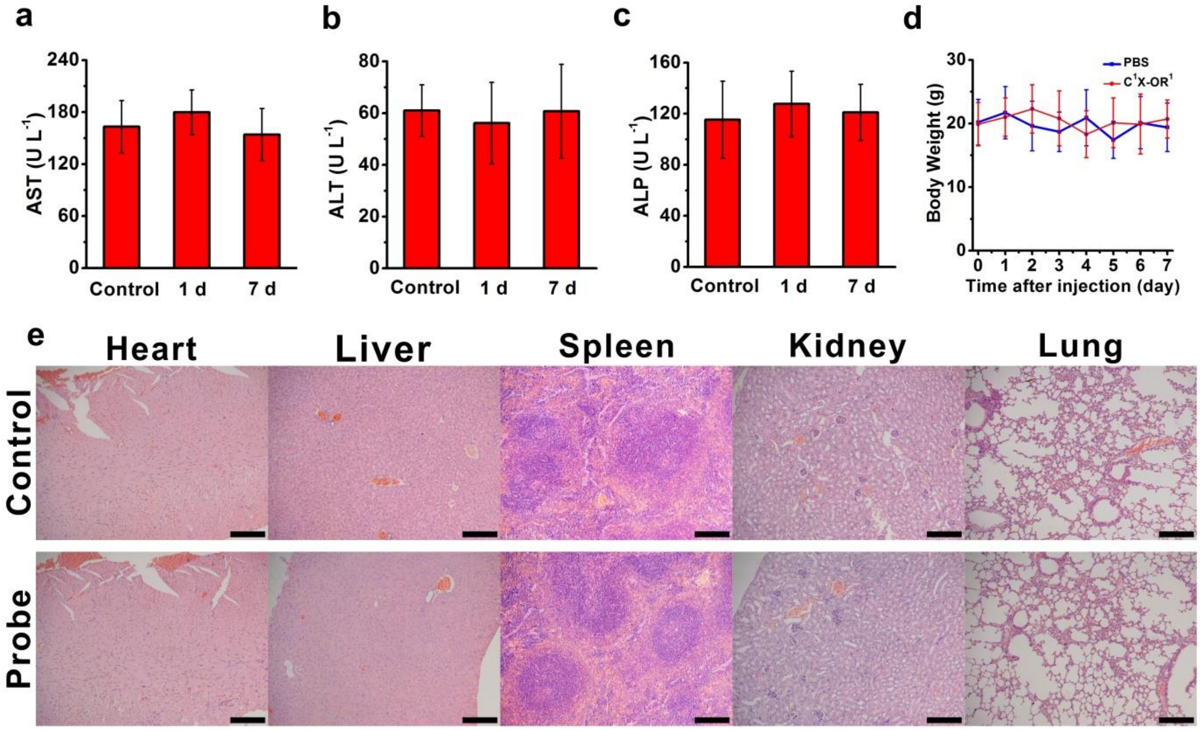

Supplementary Figure 26. Blood biochemical assays, body weight and histological analysis for C[1]X-OR[1].

Serum activities determined using ELISA kits for (**a**) aspartate aminotransferase (AST), (**b**) alanine aminotransferase (ALT), (**c**) alkaline phosphatase (ALP) for male BALB/c nude mice (8-week-old) before and after intravenous injection of C[1]X-OR[1] for different time (n = 9 per group). (**d**) Time-dependent changes in mice body weight (n = 9 per group). (**e**) Representative Histological sections (H&E staining) for main organs of the mice one day after intravenous injection of saline (control) or C[1]X-OR[1]. Scale bar: 200 μm. Columns represent means ± SD. Error bars represent the standard deviation (SD).

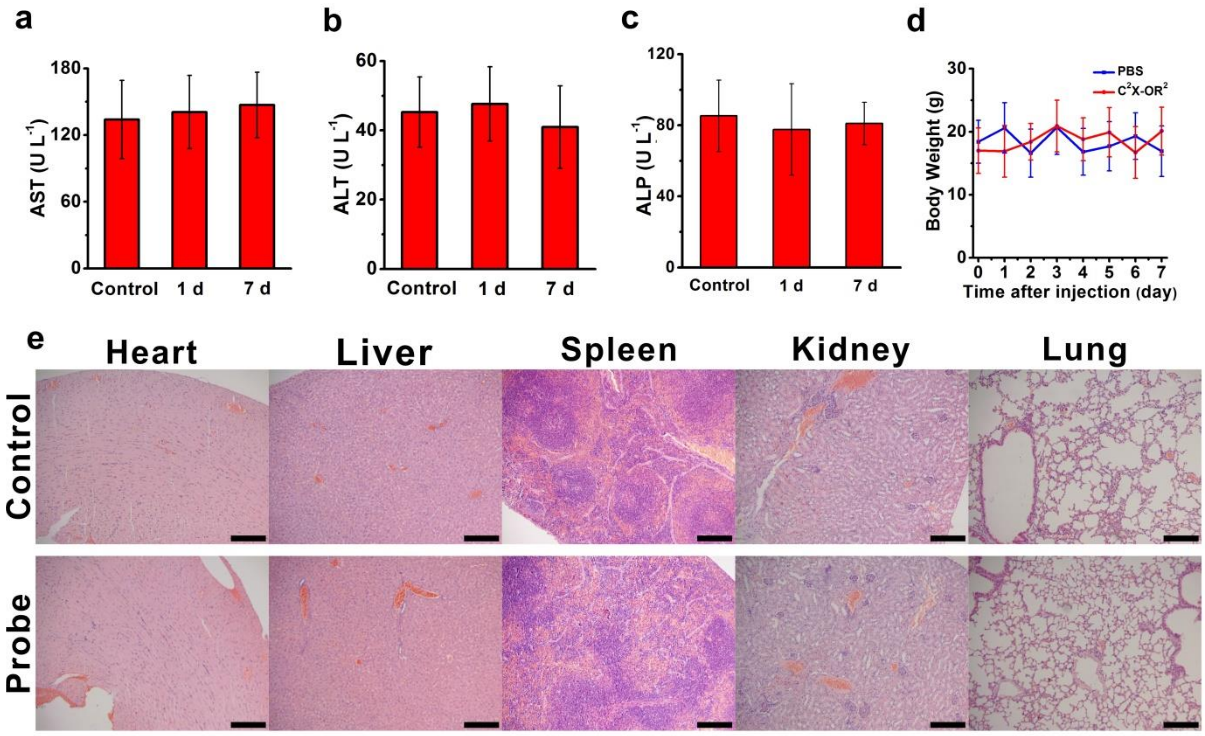

Supplementary Figure 27. Blood biochemical assays, body weight and histological analysis for $C^2X\text{-}OR^2$.

Serum activities determined using ELISA kits for (**a**) aspartate aminotransferase (AST), (**b**) alanine aminotransferase (ALT), (**c**) alkaline phosphatase (ALP); for female BALB/c nude mice (10-week-old) before and after intravenous injection of $C_2X\text{-}OR_2$ for different time (n = 9 per group). (**d**) Time-dependent changes in mice body weight (n = 9 per group). (**e**) Representative Histological sections (H&E staining) for main organs of the mice one day after intravenous injection of saline (control) or $C^2X\text{-}OR^2$. Scale bar: 200 μm. Columns represent means ± SD. Error bars represent the standard deviation (SD).

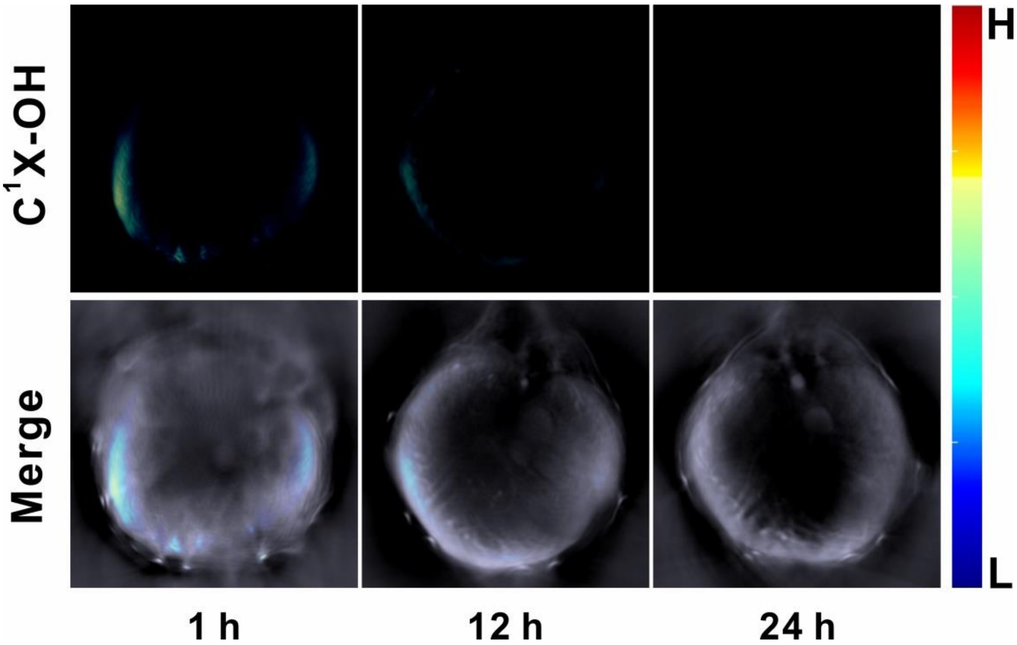

Supplementary Figure 28. Representative cross-sectional MSOT images of a mouse at varied time points upon injection of C^1X-OR^1.

The mouse was pre-treated with 300 mg kg^{-1} of APAP 12 hours in advance.

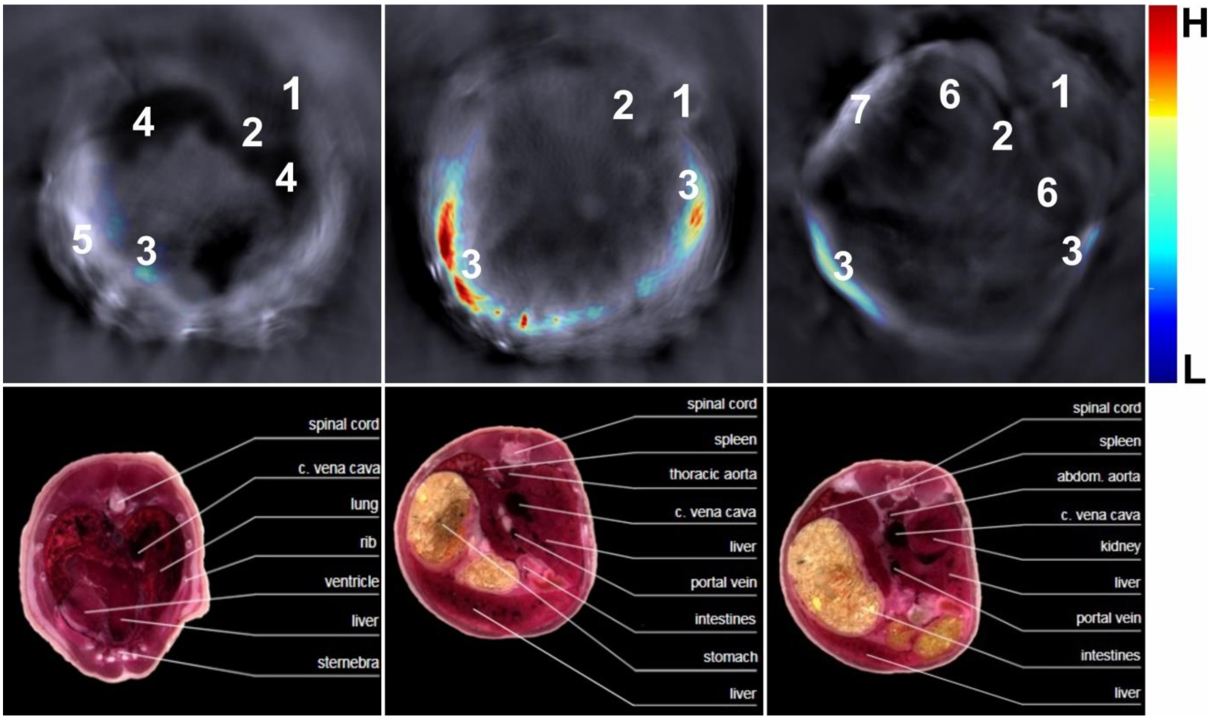

Supplementary Figure 29. Representative MSOT images at three different cross sections of a mouse. (pre-treated with 300 mg kg^{-1} of APAP 12 hours in advance) at 30 min after injection of C^1X-OR^1 (upper panel), and the corresponding anatomical cryosection images (lower panel). Organ labeling: 1: spinal cord; 2: aorta; 3: liver; 4: lung; 5: heart; 6: kidney; 7: spleen.

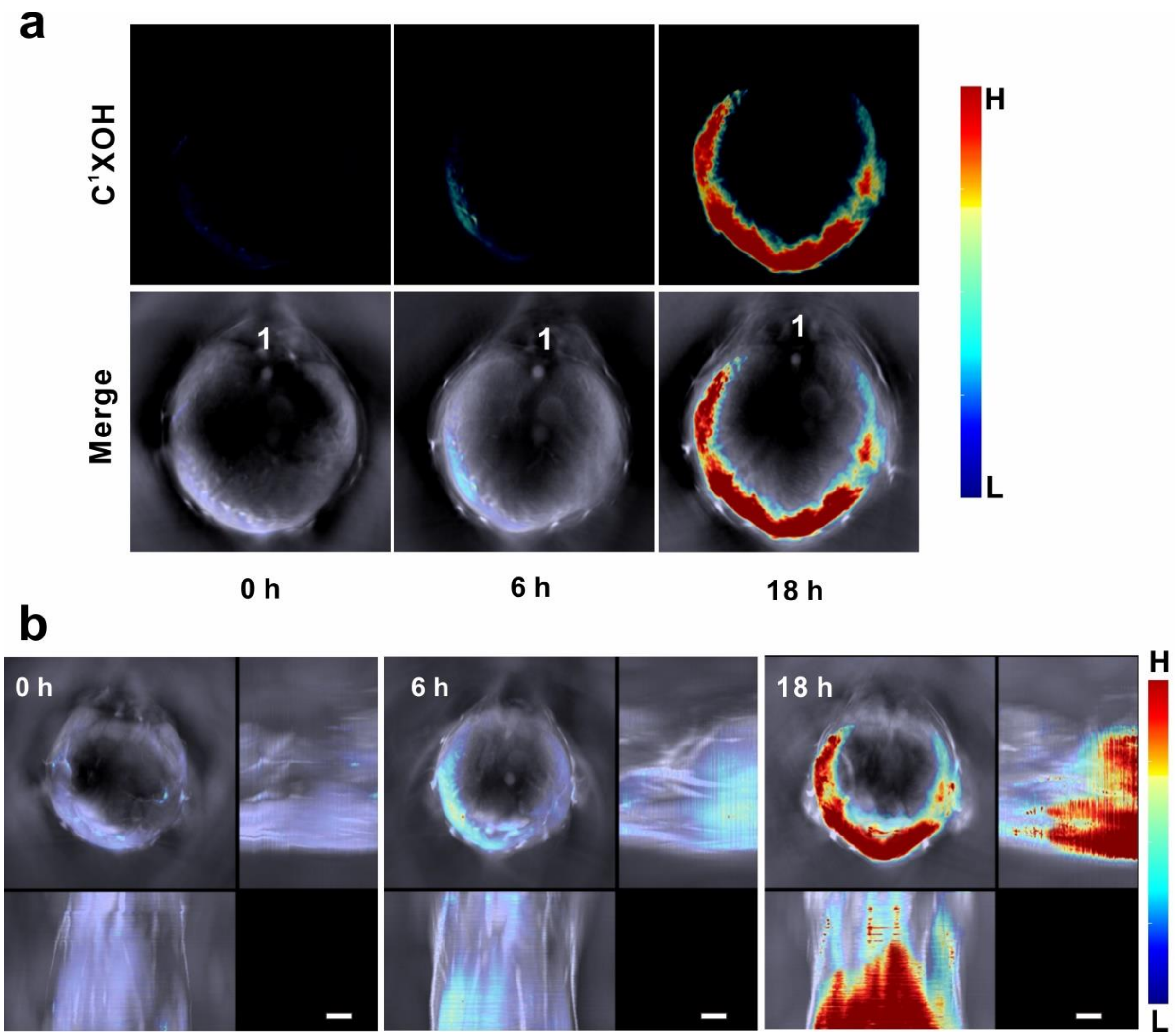

Supplementary Figure 30. Typical cross-sectional and z-stack MIP MSOT images. (a) Typical cross-sectional MSOT images; (b) z-stack MIP MSOT images; for a mouse at 0, 6 and 18 h post injection of 300 mg kg^{-1} of APAP. The mouse lies on its stomach and the number 1 indicates the location of its spinal cord.

After 18 h post injection of 300 mg kg^{-1} APAP, some mice were found dead, so the maximum time was set to 18 h.

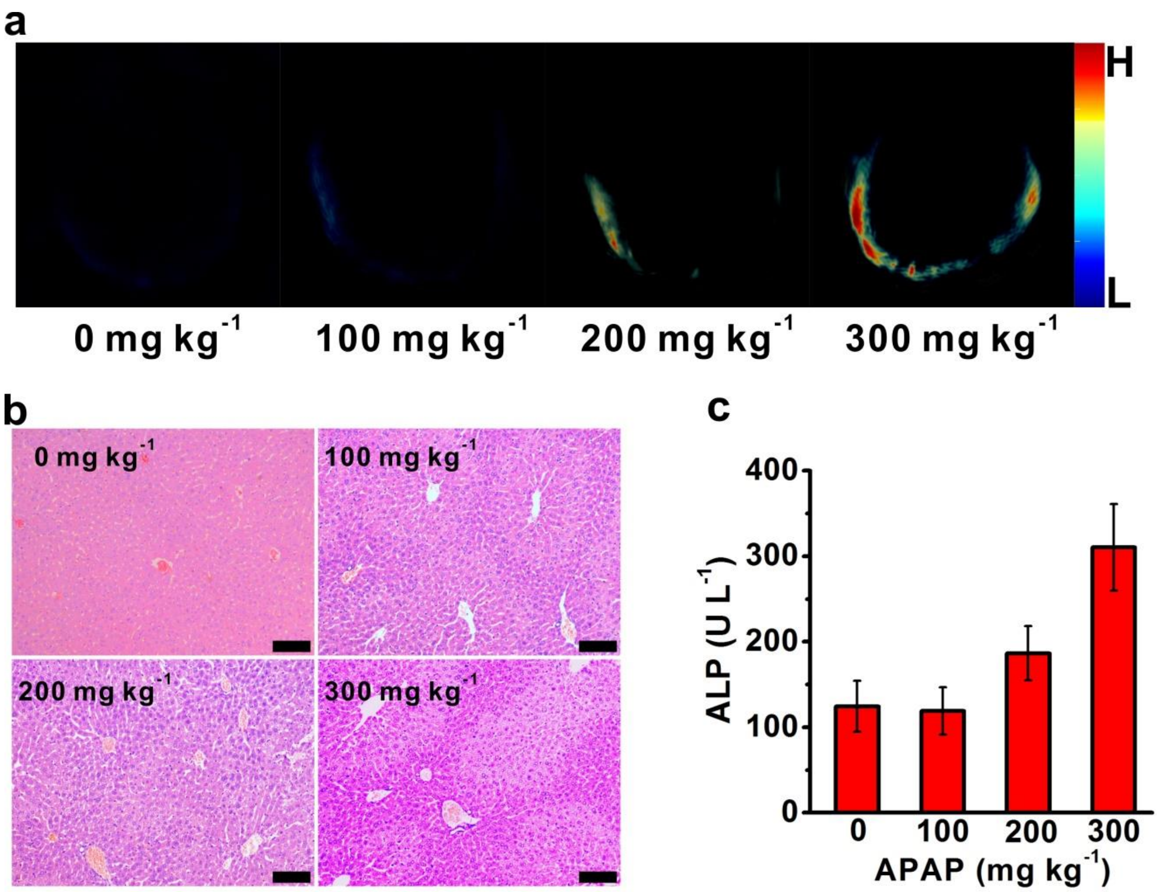

Supplementary Figure 31. Representative MSOT signals, H&E staining analyses and serum ALP levels. (**a**) Representative multispectral resolved MSOT signals for C^1X-OH (activated probe) in the mice pretreated with varied dose of APAP at 30 min upon probe injection. (**b**) Representative liver sections (H&E staining) of the mice treated with varied dose of APAP. Scale bar: 100 μm. (**c**) serum ALP levels at 12 hours upon treatment with varied dose of APAP (n = 9 per group). Columns represent means ± SD. Error bars represent the standard deviation (SD).

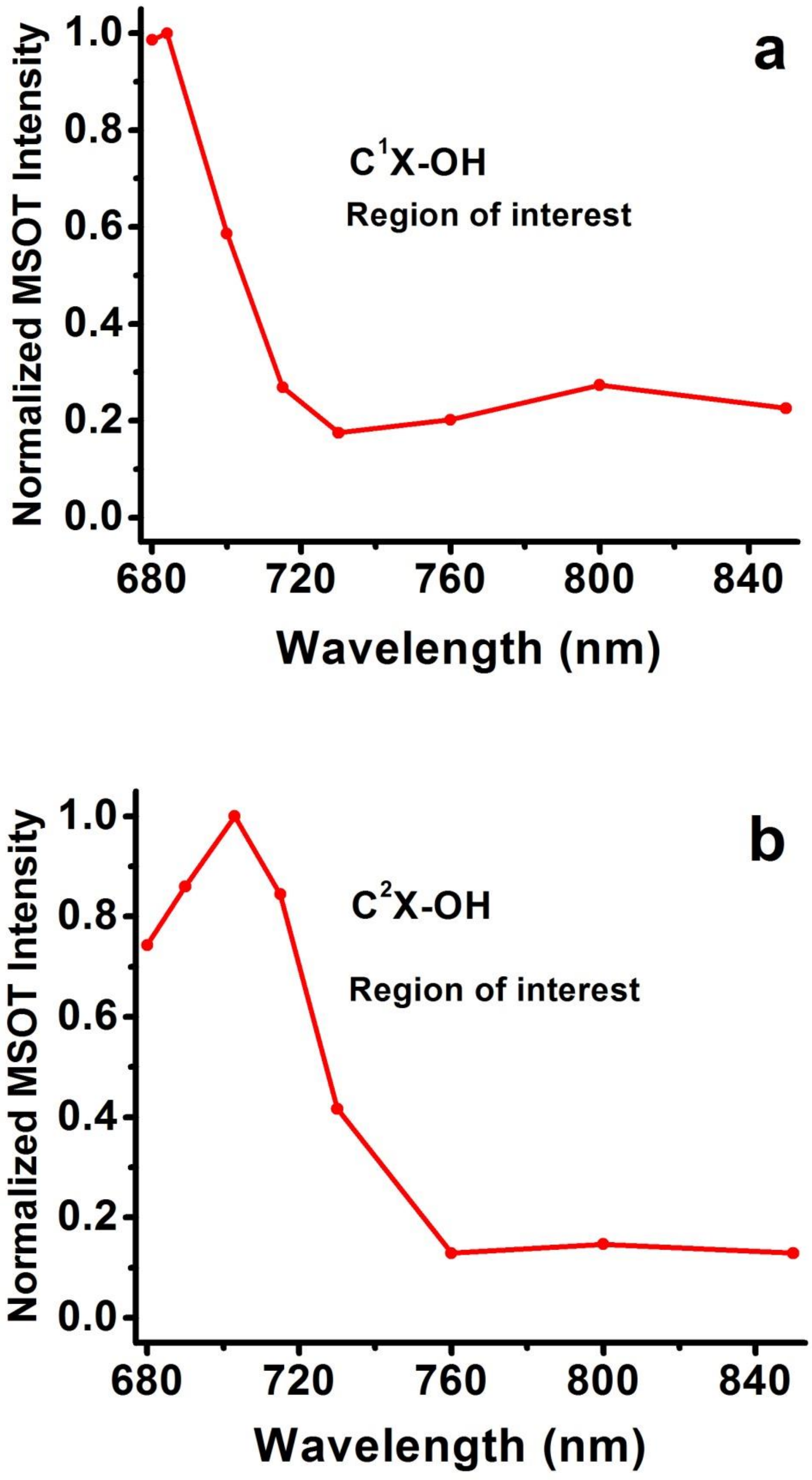

Supplementary Figure 32. Detected MSOT spectra at region of interests (liver injury area and metastatic tumor) at different wavelengths for the two probes.

The intensities were normalized to maximum values respectively.

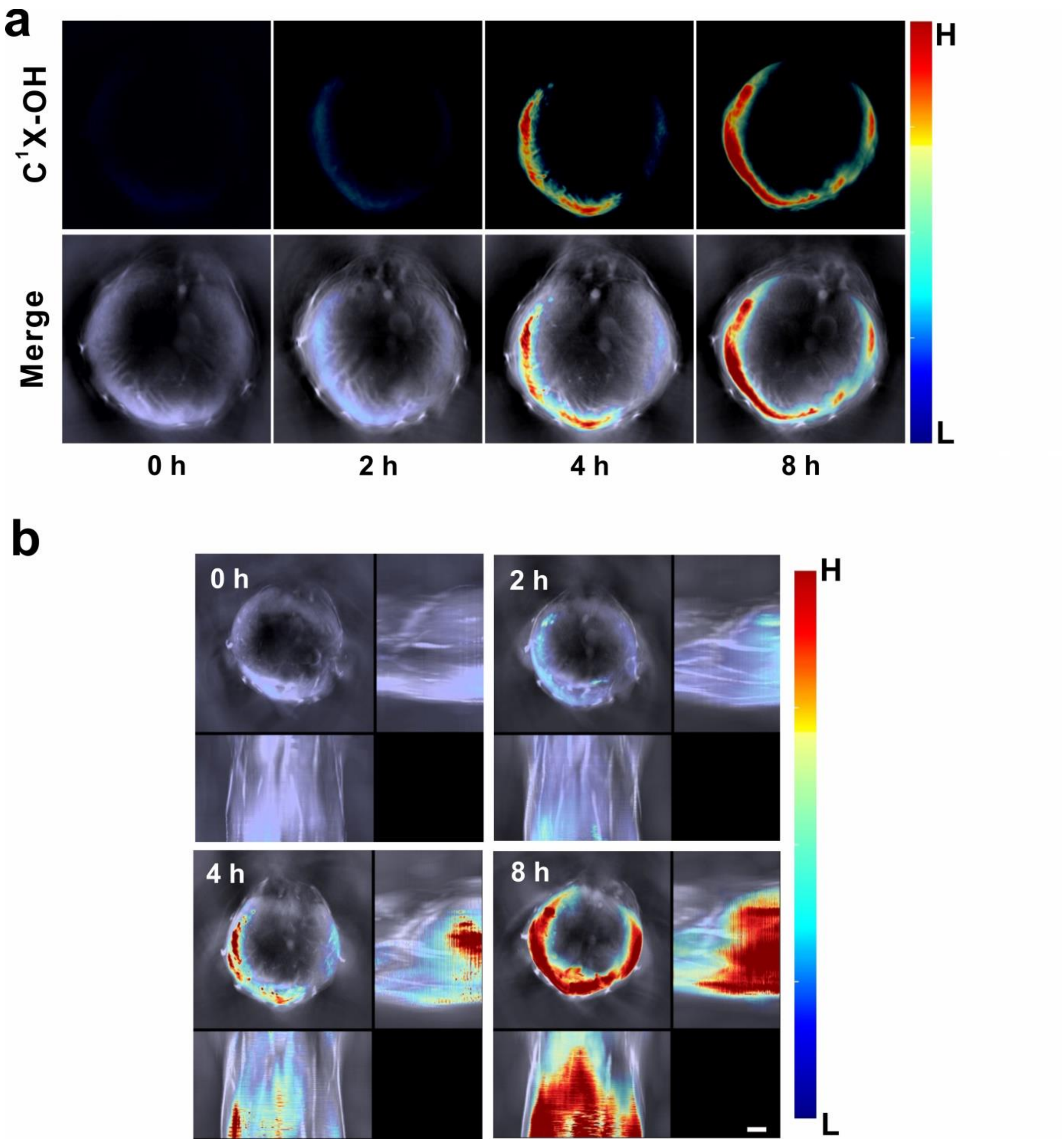

Supplementary Figure 33. Typical cross-sectional and z-stack MIP MSOT images. (a) Typical cross-sectional MSOT images for a mouse; (b) z-stack MIP MSOT images; at 0, 2, 4, and 8 h after combined treatment of 700 mg kg^{-1} of D-galactosamine and 20 μg kg^{-1} of TNF-α. For the drug treatment, D-galactosamine was intraperitoneally injected followed by the i.v. injection of TNF-α 5 min later.

At 8 h post injection of the drug, some mice were found dead, so the maximum time was set to 8 h.

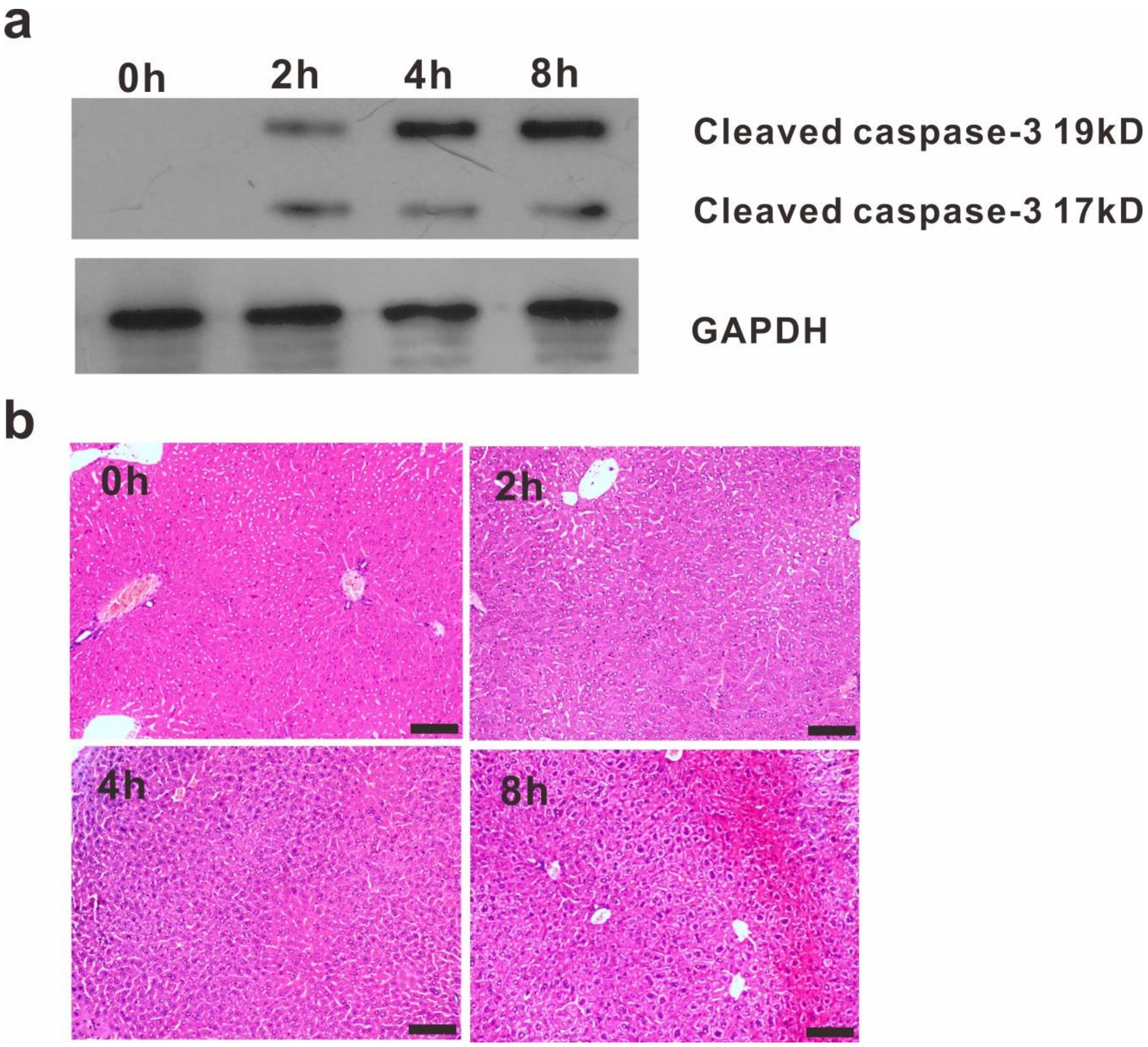

Supplementary Figure 34. Typical Western blotting analyses and H&E staining analyses. (a) Typical Western blotting analyses illustrating the level of cleaved caspase-3 in liver tissue lysis for the mice at 0, 2, 4, and 8 h after combined treatment of 700 mg kg^{-1} of D-galactosamine and 20 μg kg^{-1} of TNF-α. (b) Typical H&E staining for liver sections for the mice at 0, 2, 4, and 8 h after combined treatment of 700 mg kg^{-1} of D-galactosamine and 20 μg kg^{-1} of TNF-α. Scale bar: 100 μm.

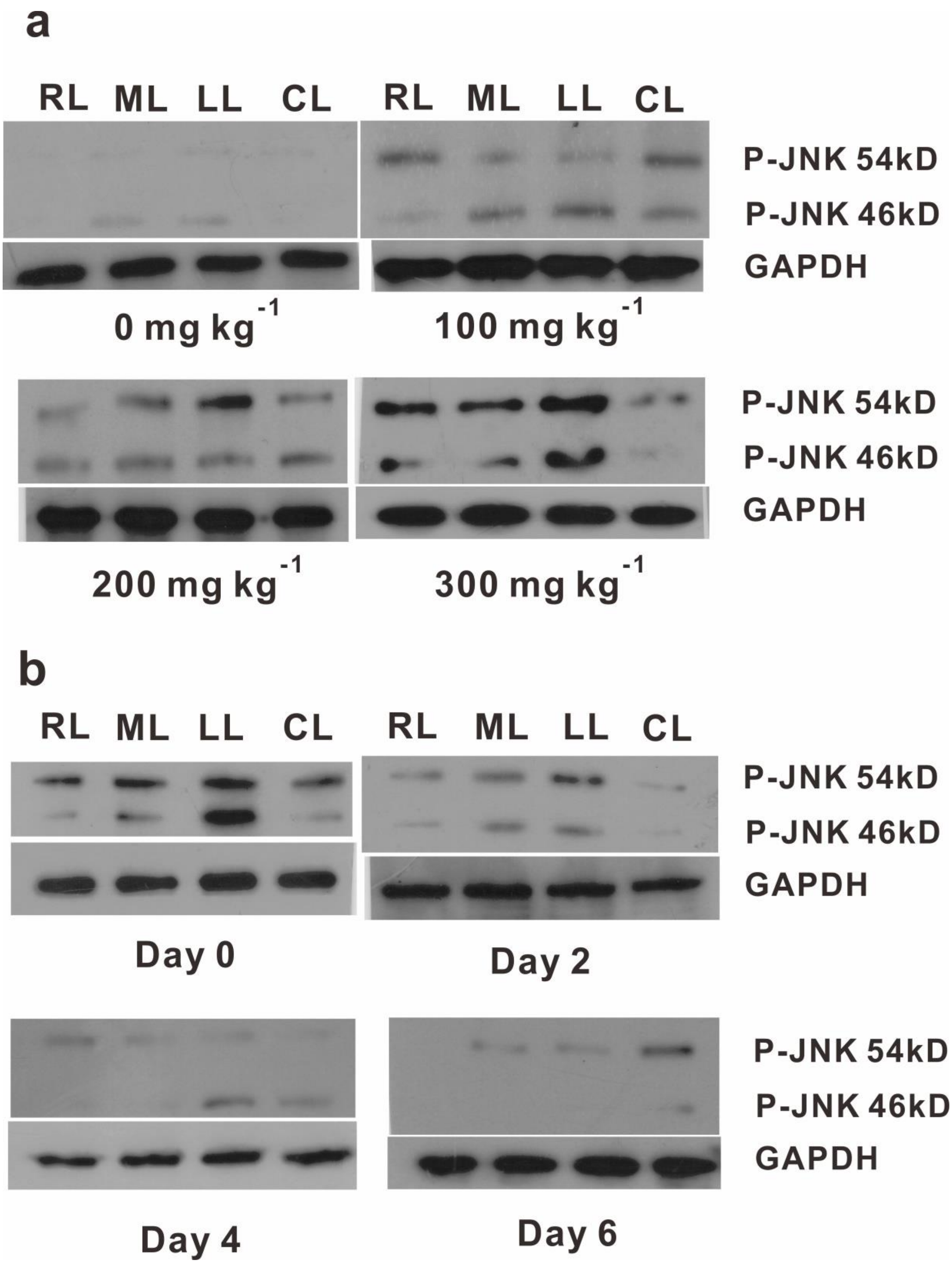

Supplementary Figure 35. Typical Western blotting analyses illustrating the level of phosphorylated JNK (P-JNK) in liver tissue lysis from right lobe (RL), left lobe (LL), medial lobe (ML) or caudate lobe (CL), respectively. (a) Mice at 12 h upon treatment with varied doses of APAP. (b) Liver-injured mice (overdosed with 300 mg kg^{-1} of APAP) upon treatment with N-acetylcysteine for varied days.

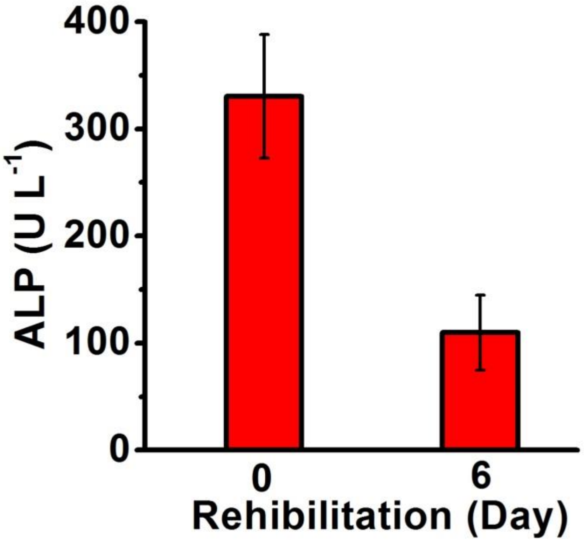

Supplementary Figure 36. Serum ALP level for the mice upon N-acetylcysteine (150 mg kg^{-1}) treatment for 0 and 6 days. Columns represent means ± SD. Error bars represent the standard deviation (SD) (n = 9 per group).

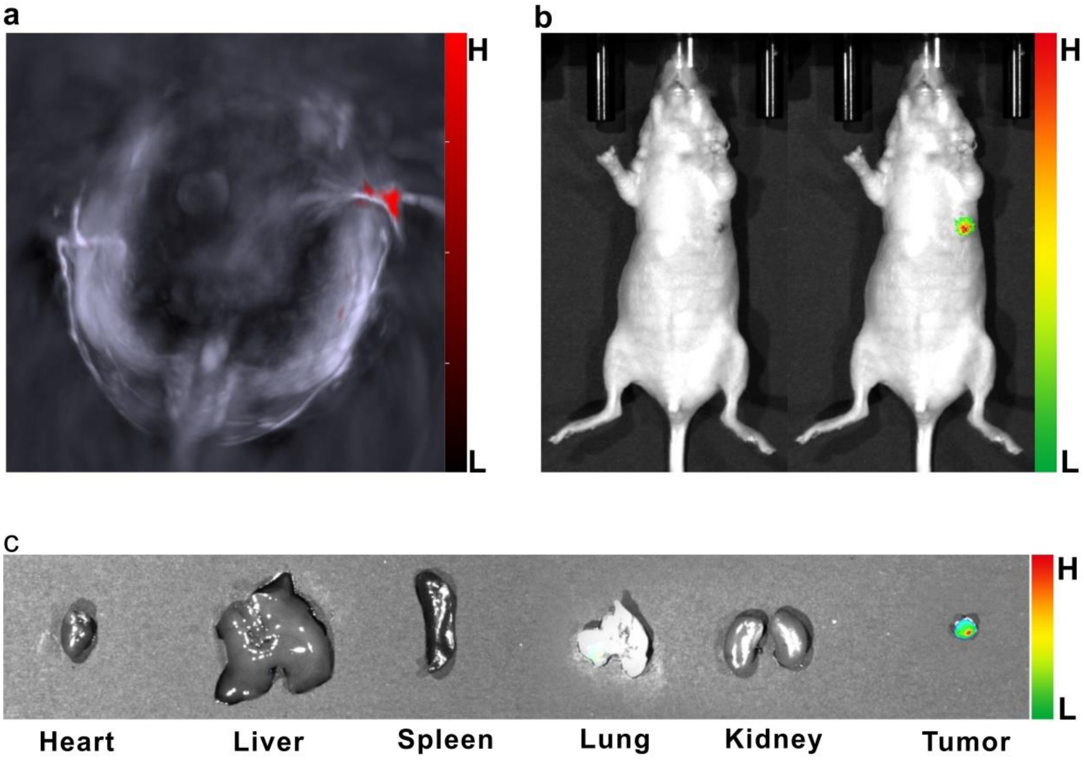

Supplementary Figure 37. Representative cross-sectional MSOT image and fluorescent images. (**a**) Representative cross-sectional MSOT image of the mice at 3 weeks upon injection of OVCAR 3 cancer cells subcutaneously. (**b**) Representative fluorescent images of the mice at 3 weeks upon injection of OVCAR 3 cancer cells subcutaneously. (**c**) Representative fluorescent images of excised organs and tumor at 30 min after intratumoral injection of C^2X-OR^2.

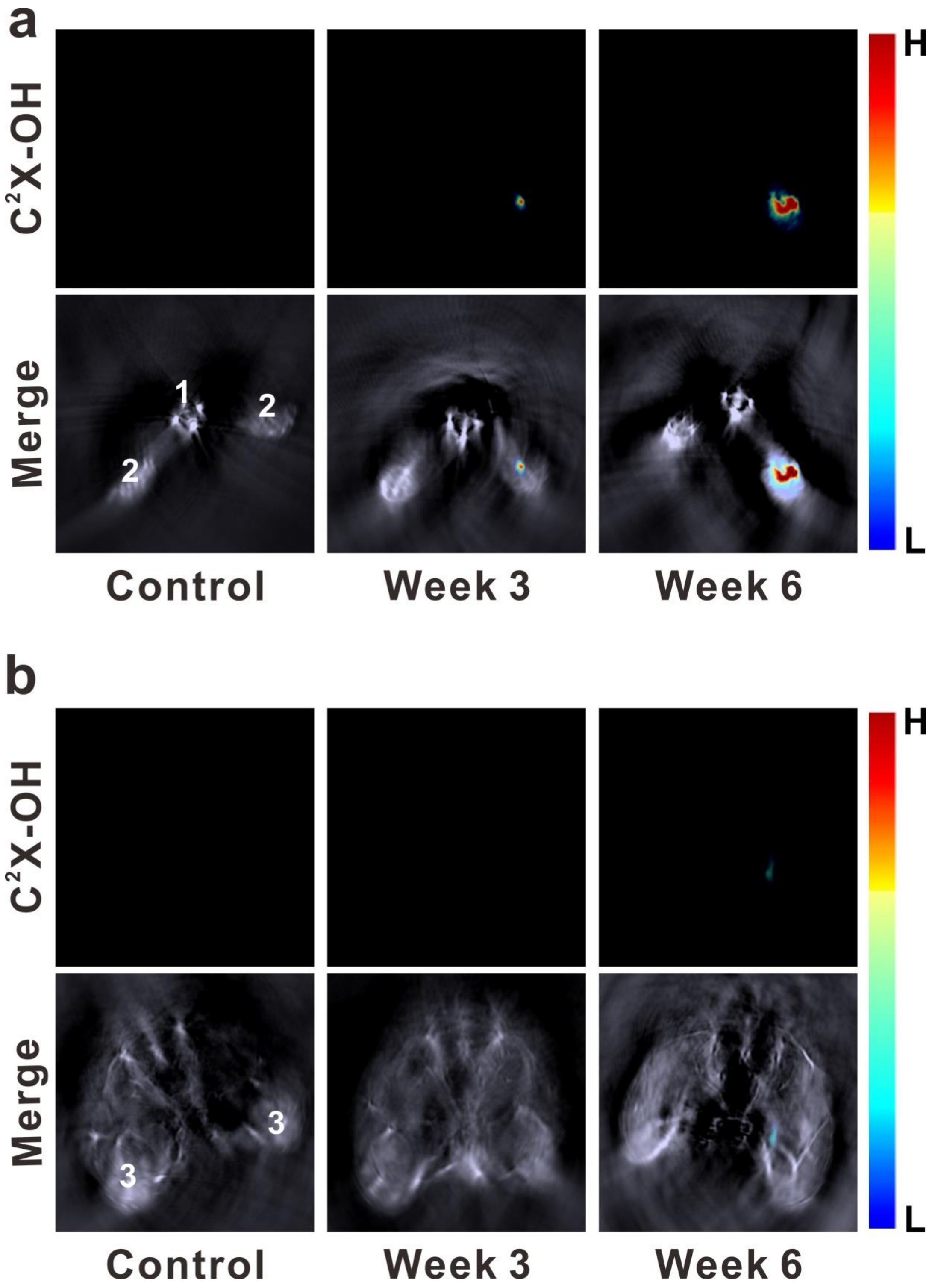

Supplementary Figure 38. Two cross-sectional images (for lymphatic metastasis) for the control and the mice at 3 or 6 weeks upon injection of ovarian cancer cells (SKOV3) at right hind footpad. (a) Cross-section at footpads/tail. (b) Cross-section at popliteal position. Labeling: 1. Tail; 2. Paw; 3. Hind thighs. The corresponding Z-stack 3D images with their data obtained from dozens of cross-sections are given in Fig. 5e in the main text.

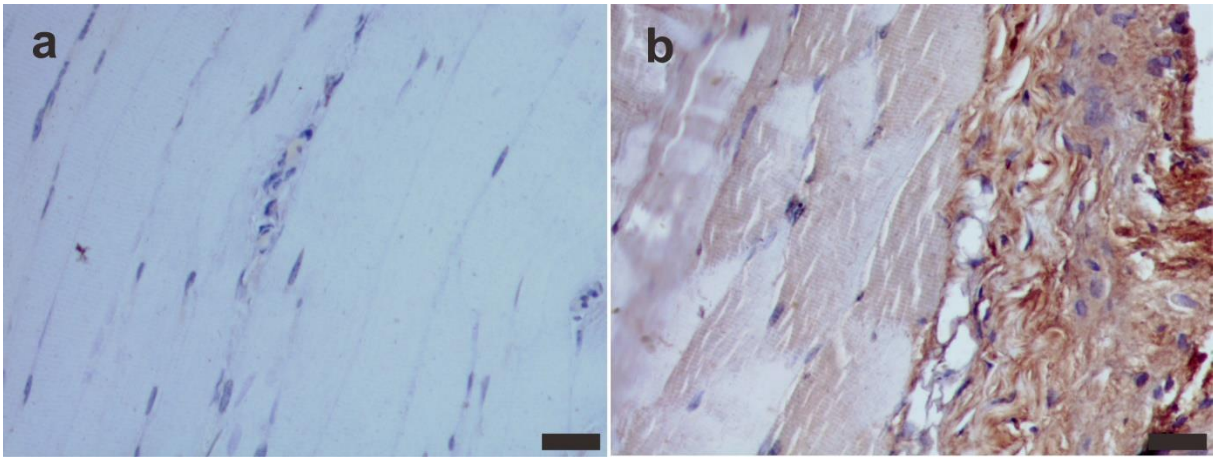

Supplementary Figure 39. Immunohistochemical analysis of CD206. (a) in popliteal tissue from a healthy mouse; (b) in popliteal tumor adjacent tissue from a tumor-bearing mouse. The brown color indicates the high expression of CD206.

Previous studies indicate that, M2 macrophages play an important role in tumor metastasis,[5] and accumulation of M2 macrophages correlate with ovarian cancer metastases.[6] Macrophage mannose receptor (MMR), or CD206, is a specific M2 macrophage marker and highly expressed on M2 macrophages. Thus, detection of CD206 within the tumor microenvironment can provide indication for tumor metastasis. This figure shows the IHC analysis of CD206 in popliteal tissue from a healthy mouse (the control) and in popliteal tumor adjacent tissue from a tumor-bearing mouse. One can find that, the metastatic tumor adjacent tissue exhibited significant expression of CD206, indicating the CD206-positive macrophage level was increased in the tumor adjacent tissues at the popliteal position. This IHC analysis proves the occurrence of lymphatic metastasis in popliteal position, which has been detected and mapped by MSOT.

The procedure of the IHC analysis is presented in Supplementary methods.

Supplementary Methods

Materials. Cyclohexanone, 1,2,3,3-tetramethyl-3H-indolium iodide, resorcinol, diethyl chlorophosphate, bromotrimethylsilane, tetra-O-acetyl-α-D-galactopyranosyl-1-bromide, 1,1,2-Trimethylbenz[e]indole, cholic acid, N-(3-Dimethylaminopropyl)-N'-ethylcarbodiimide hydrochloride crystalline (EDC·HCl), N-Hydroxysulfosuccinimide sodium salt (Sulfo-NHS) and D-galactosamine were obtained from Sigma-Aldrich and used without further purification. Recombinant human tumor necrosis factor-α was purchased from Sangon Biotech (shanghai) Co. Ltd. Sodium acetate, silver carbonate, sodium methoxide, cysteine (Cys), homocysteine (Hcy), glutathione (GSH), ascorbic acid (AA), Hydrogen peroxide solution (H_2O_2), N,N-dimethylformamide (DMF for HPLC grade), dichloromethane (DCM for HPLC grade), acetonitrile (MeCN for HPLC grade), methanol (MeOH for HPLC grade), tetrahydrofuran (THF for HPLC grade) and dimethylsulfoxide (DMSO for HPLC grade) were purchased from Aladdin. Phosphorus oxychloride was obtained from Xiya Reagent. 1,2-Dipalmitoyl-sn-glycero-3-phosphocholine (DPPC), 1,2-distearoyl-sn-glycero-3-phosphocholine (DSPC) and 1,2-distearoyl-sn-glycero-3-phosphoethanolamine-N-[amino(polyethylene glycol)-2000] (DSPE-PEG_{2000}-NH_2) were obtained from Tokyo Chemical Industry Co. Ltd. Alkaline phosphatase (ALP), β-Galactosidase (Gal), acetylcholinesterase (AchE), glutamyl transferase (GGT), carboxylesterase (CaE) and nitroreductase (NTR) were obtained from Sigma-Aldrich. Elisa Kits of alkaline phosphatase (ALP), aspartate transaminase (AST), alanine aminotransferase (ALT) and CA125 were purchased from Shanghai Enzyme-linked Biotechnology Co. Ltd. Potassium carbonate, sodium sulfate and all other solvents were analytical-grade and obtained from Guangzhou Chemical Reagent Factory. The water used throughout the experiments was triple-distilled water.

Measurements. ^1H NMR (600 MHz), ^{13}C NMR (600 MHz) and ^{31}P NMR (600 MHz) spectra were measured with Bruker Avance NMR Spectrometer. ESI mass spectra were obtained by using Bruker Esquire HCT Plus mass spectrometer. High-resolution mass spectrometry was conducted with AB Sciex Triple TOF 5600+ mass spectrometer or Sciex X500R. The particle size and distribution was determined through dynamic light scattering (DLS) on a Malvern Nano-ZS90 particle size analyzer at a fixed angle of 90° at 25 ℃. Transmission electronic microscopy (TEM) experiments were performed by dropping a drop of the solution onto the ultra-thin carbon film and observation was carried out on a JEM-1400 transmission electron microscopy. UV-vis spectra were measured on a Hitachi U-3010 UV-vis spectrophotometer. Fluorescence spectra were measured by using a Hitachi F-4600 fluorescence spectrophotometer. HPLC data were acquired from Agilent 1260 Infinity liquid chromatograph (with DAD). Fluorescence microscopic images were obtained using an Olympus IX 71 with a DP72 color CCD. Flow cytometry were performed on a BD CS6 flow cytometer. Photoacoustic imaging was performed on an inVision128 multispectral optoacoustic tomographic (MSOT) imaging system (iThera Medical GmbH) and fluorescent imaging was performed on an AMI small animal fluorescence imaging system (Spectral Instruments Imaging Co.).

Synthesis of Compound 1. Freshly distilled dimethylformamide (20 mL, 273 mmol) was added into a 250 mL two-neck round flask under nitrogen atmosphere. Afterwards, phosphorus oxychloride (20 mL, 131 mmol) dissolved in 20 mL dichloromethane was added dropwise within 20 min at 0 °C. After being stirred at room temperature for 30 min, cyclohexanone (5.0 g, 51 mmol) was slowly added into the mixture via a syringe. The solution was refluxed with vigorously stirring for 1.5 h at 80 °C. After that, the resulting mixture was cooled down, poured into ice-cold water and

allowed to stand in a refrigerator overnight. The suspension was filtered and precipitate was collected and dried in vacuum as yellow solid (5.2 g, yield: 59%).

Synthesis of Compound 2 (C^1Y). Compound 1 (0.86 g, 5 mmol) and 1,2,3,3-tetramethyl-3H-indolium iodide (3.01 g, 10 mmol) were dissolved in acetic anhydride (40 mL) followed by addition of sodium acetate (0.82 g, 10 mmol). The mixture was refluxed at 130 °C for 1 h under nitrogen atmosphere. Afterwards, the solvent was removed by vacuum-rotary evaporation procedure, and the resulting green solid mixture was purified by silica gel chromatography with dichloromethane/methanol (20:1) as eluent to obtain 2 as a dark green solid (2.8 g, yield: 91%). ^1H NMR (600 MHz, DMSO-d_6): δ 8.23 (d, 2H, J = 18 Hz), 7.62 (d, 2H, J = 12 Hz), 7.46-7.43 (m, 4H), 7.31-7.27 (m, 2H), 6.29 (d, 2H, J = 18 Hz), 3.69 (s, 6H), 2.72 (t, 4H, J = 15 Hz), 1.89-1.83 (m, 2H), 1.67 (s, 12H). ESI-MS (m/z): 483.2 $[M]^+$

Synthesis of Compound 3 (C^2Y). 1,1,2,3-Tetramethyl-1H-benz[e]indolium iodide (C^2) was prepared according to the literature method[4]. Compound 1 (0.43 g, 2.5 mmol) and 1,1,2,3-tetramethyl-1H-benz[e]indolium iodide (1.76g 5 mmol) were dissolved in acetic anhydride (20 mL) followed by addition of sodium acetate (0.41 g, 5 mmol). The mixture was refluxed at 130 °C for 1 h under nitrogen atmosphere. Afterwards, the solvent was removed by vacuum rotary evaporation procedure, and the resulting green solid mixture was purified by silica gel chromatography with dichloromethane/methanol (30:1) as eluent to obtain 2 as a dark green solid (1.5 g, yield: 85%). ^1H NMR (600 MHz, DMSO-d_6): δ 8.35 (d, 2H, J = 1.8 Hz), 8.29 (d, 2H, J = 8.4 Hz), 8.10 (d, 2H, J = 8 Hz), 8.07 (d, 2H, J = 8.4 Hz), 7.67 (t, 2H, J = 15.6 Hz), 7.53 (t, 2H, J = 13.8 Hz), 7.50-7.46 (m, 2H), 6.34 (d, 2H, J = 14.4 Hz), 3.82 (s, 6H). 2.80-2.73 (m, 4H), 1.96 (s, 12H),

1.91 (s, 2H). ESI-MS (m/z): 582.7 $[M]^+$

Synthesis of Compound 4 (C^1X-OH). Into a 100 mL two-neck round-bottom flask, resorcinol (0.88 g, 8 mmol) was dissolved in anhydrous acetonitrile (30 mL), and then potassium carbonate (1.1 g, 8mmol) was added. After being stirred at room temperature for 30 min, C^1Y (2.44 g, 4 mmol) dissolved in acetonitrile (20 mL) was added dropwise. The mixture was heated at 55 °C for 4 h under nitrogen atmosphere. The solvent was removed by vacuum-rotary evaporation procedure, and the crude product was purified by silica gel column chromatography with dichloromethane/methanol (20:1) as eluent to give a greenish blue solid (1.44 g, yield: 70%). ^1H NMR (600 MHz, DMSO-d_6): δ 8.53 (d, 1H, J = 15 Hz), 7.73 (d, 1H, J = 7.2 Hz), 7.59 (d, 1H, J = 7.8 Hz), 7.53-7.49 (m, 2H), 7.47 (d, 1H, J = 8.4 Hz), 7.40 (d, 1H, J = 15 Hz), 6.87 (d, 1H, J = 1.8 Hz), 6.82 (m, 1H), 6.43 (d, 1H, J = 14.4 Hz). 3.81 (s, 3H), 2.72 (t, 2H, J = 11.4 Hz), 2.67 (t, 2H, J = 12 Hz), 1.80-1.85 (m, 2H), 1.73 (s, 6H). ESI-MS (m/z): 383.20 $[M]^+$

Synthesis of Compound 5. Into a 50 mL round-bottom flask was added of Compound 4 (1.0 g, 1 mmol) and potassium carbonate (0.68 g, 5 mmol) in of anhydrous acetonitrile (20 mL). The mixture was stirred at room temperature for 30 min. Afterwards, a solution of diethyl chlorophosphate (3.44 g, 20 mmol) in acetonitrile (10 mL) was added to the above mixture via a syringe, and the reaction mixture was heated at 40 ℃ overnight. The solvent was removed by evaporation, and the residue was purified by silica gel column chromatography using dichloromethane/methanol (v/v 30:1). After being dried in vacuum oven, the dark blue solid was obtained (1.08 g, yield: 83%). ^1H NMR (600 MHz, DMSO-d_6): δ 8.56 (d, 1H, J = 15.6 Hz), 7.79 (d, 1H, J = 7.8 Hz), 7.73 (d, 1H, J = 7.8 Hz), 7.62-7.54 (m, 2H), 7.51 (d, 1H, J = 15.6 Hz), 7.39-7.37 (m, 1H), 7.21-7.18 (m, 1H), 6.66 (d,

1H, J = 15 Hz), 5.32 (d, 1H, J = 9.6 Hz). 4.25-4.19 (m, 4H), 3.94 (s, 3H), 2.74 (t, 2H, J = 12 Hz), 2.68 (t, 2H, J = 12 Hz), 1.85-1.82 (m, 2H), 1.76 (s, 6H), 1.23 (s, 6H). ESI-MS (m/z): 519.7 $[M]^+$

Synthesis of Compound 6 (C^1X-OR^1). Compound 5 (0.64 mg, 1 mmol) was dissolved with anhydrous acetonitrile (15 mL) in a 50 mL round-bottom flask and the reaction flask was purged with dry nitrogen three times. After that, bromotrimethylsilane (3.06 g, 20 mmol) was injected via a syringe. The mixture was stirred at room temperature for 2 days. Eventually, 6 mL methanol was added to quench the reaction. The resulting mixture was stirred for another day. The reaction solvent was removed under reduced pressure, and the resulting residue was purified by silica gel column chromatography using dichloromethane/methanol (v/v 15:1) to yield C^1X-OR^1 as a dark blue solid (0.2 g, yield: 45%). ^1H NMR (600 MHz, CD_3OD): δ 8.67 (d, 1H, J = 16.2 Hz), 7.56 (d, 1H, J = 7.8 Hz), 7.46-7.42 (m, 1H), 7.39-7.34 (m, 2H), 7.33-7.30 (m, 1H), 7.28-7.26 (m, 1H), 7.07 (d, 1H, J = 5.6 Hz), 6.44 (d, 1H, J = 13.8 Hz), 5.25-5.23 (m, 1H). 3.79 (s, 3H), 2.69 (t, 2H, J = 11.4 Hz), 2.63 (t, 2H, J = 11.4 Hz), 1.87-1.82 (m, 2H), 1.73 (s, 6H). ^{13}C NMR (600 MHz, CD_3OD): δ 118.00, 161.27, 155.85, 153.61, 146.25, 142.47, 141.04, 132.59, 128.94, 128.48, 127.39, 122.53, 118.54, 117.77, 114.33, 112.59, 107.09, 107.01, 103.89, 50.86, 29.50, 28.74, 26.85, 23.60, 20.31. ^{31}P NMR (600 MHz, CD_3OD): δ -5.04. MS (m/z): 464.1620 $[M]^+$

Synthesis of Compound 7 (C^2X-OH). Into a 100 mL two-neck round-bottom flask, resorcinol (0.22g, 2 mmol) was dissolved in anhydrous acetonitrile (10 mL), and then potassium carbonate (0.28 g, 2 mmol) was added. After being stirred at room temperature for 30 min, C^2Y (1.42 g, 2 mmol) dissolved in acetonitrile (5 mL) was added dropwise. The mixture was heated at 55 °C for 4 h under nitrogen atmosphere. The solvent was removed by vacuum-rotary evaporation procedure,

and the crude product was purified by silica gel column chromatography with dichloromethane/methanol (20:1) as eluent to give a greenish blue solid (0.63 g, yield: 56%). ^{1}H NMR (600 MHz, DMSO-d_6): δ 8.21 (d, 1H, J = 8 Hz), 8.08 (d, 1H, J = 13.2 Hz), 7.96-7.94 (m, 1H), 7.94-7.92 (m, 1H), 7.62-7.60 (m, 1H), 7.52 (t, 1H, J = 7.8 Hz), 7.51 (t, 1H, J = 7.8 Hz), 7.36 (d, 1H, J = 7.2 Hz), 7.34 (d, 1H, J = 8 Hz). 6.44 (d, 1H, J = 8 Hz), 6.20-6.18 (m, 1H), 5.76 (d, 1H, J = 2.4 Hz), 3.50 (s, 3H), 2.68-2.60 (m, 4H), 1.94 (s, 6H), 1.84-1.78 (m, 2H). ESI-MS (m/z): 433.4 $[M]^+$

Synthesis of Compound 8. C^2X-OH (0.56 g, 1 mmol) was dissolved in dry acetonitrile (30 mL) and then sodium sulfate (0.36 g, 2.5 mmol) and silver carbonate (1.38 g, 5 mmol) was added. The mixture was stirred for 0.5 h at room temperature. Afterwards, tetra-O-acetyl-α-D-galactopyranosyl-1-bromide (0.62 g, 1.5 mmol) was added into the solution and the mixture was stirred overnight under N_2 atmosphere. The reaction solvent was removed under reduced pressure, and the resulting residue was purified by silica gel column chromatography using dichloromethane/methanol (v/v 15:1) and a dark blue solid was obtained (0.58 g, yield: 65%). ^{1}H NMR (600 MHz, DMSO-d_6): δ 8.69 (d, 1H, J = 15 Hz), 8.39 (d, 1H, J = 8.4 Hz), 8.22 (d, 1H, J = 8 Hz), 8.17 (d, 1H, J = 8.4 Hz), 8.00 (d, 1H, J = 8 Hz), 7.77 (d, 1H, J = 15.6 Hz), 7.66 (t, 1H, J = 15 Hz), 7.57 (t, 1H, J = 8 Hz), 7.40-7.38 (m, 1H). 7.15-7.10 (m, 1H), 7.04 (m, 1H), 6.66 (d, 1H, J = 15 Hz), 5.85-5.75 (m, 1H), 5.40 (d, 1H, J = 3 Hz), 5.37-5.32 (m, 1H), 5.31-5.26 (m, 1H), 4.56 (m, 1H), 4.10 (m, 2H), 4.06 (s, 3H), 2.80-2.65 (m, 4H), 2.20-2.13 (m, 3H), 2.10-2.06 (m, 3H), 2.04-2.00 (m, 6H), 2.00-1.97 (m, 6H), 1.96-1.90 (m, 2H). ESI-MS (m/z): 764.0 $[M]^+$

Synthesis of Compound 9 (C^2X-OR^2). Compound 8 (446 mg, 0.5 mmol) was dissolved in dry methanol (20 mL), and sodium methoxide (108 mg, 2.0 mmol) in dry methanol (5 mL) was added

dropwise to the solution, and the solution was stirred for 3 h at room temperature. When reaction was completed, the reaction mixture was neutralized by adding Amberlite IR-120 plus (H^+) until pH value was about 7. Purification by silica gel chromatography with dichloromethane/methanol (10:1) provided $C_2X\text{-}OR_2$, a dark blue solid (200 mg, yield: 55%). ^1H NMR (600 MHz, CD_3OD): δ 8.86 (d, 1H, J = 16.8 Hz), 8.41 (d, 1H, J = 8.4 Hz), 8.14 (d, 1H, J = 8 Hz), 8.07 (d, 1H, J = 8.4 Hz), 7.81 (d, 1H, J = 8 Hz), 7.74 (d, 1H, J = 15 Hz), 7.61 (t, 1H, J = 15 Hz), 7.45 (t, 1H, J = 8.4 Hz), 7.33-7.29 (m, 1H). 7.29-7.26 (m, 1H), 7.10-7.06 (m, 1H), 6.57 (d, 1H, J = 15 Hz) 5.01 (d, 1H, J = 7.8 Hz), 4.00 (s, 3H), 3.98 (d, 1H, J = 3 Hz), 3.89-3.85 (m, 3H), 3.68-3.61 (m, 2H), 2.80-2.72 (m, 4H), 2.15-2.05 (m, 6H), 1.98-1.92 (m, 2H). ^{13}C NMR (600 MHz, CD_3OD): δ 180.05, 160.61, 153.96, 145.06, 139.62, 136.03, 133.08, 132.20, 130.79, 129.80, 128.30, 127.88, 127.82, 127.56, 125.98, 122.53, 116.85, 114.84, 114.06, 111.36, 103.83, 103.39, 101.77, 76.34, 73.51, 70.78, 68.90, 61.37, 52.50, 32.02, 28.76, 26.55, 26.39, 23.59, 20.27. MS (m/z): 596.2642 $[M]^+$.

Synthesis of Compound 10 (DSPE-PEG-ChA). Cholic acid (41 mg, 0.1 mmol), EDC•HCl (38 mg, 0.2 mmol) and Sulfo-NHS (43 mg, 0.2 mmol) were dissolved in dry N,N-dimethylformamide (2 mL) under a nitrogen atmosphere. The mixture was stirred at room temperature for 2 hours. Then 1,2-distearoyl-sn-glycero- 3-phosphoethanolamine-N-[amino(polyethylene glycol)-2000] (27.5 mg, 0.01 mmol) was introduced and the reaction mixture was further stirred at room temperature for 2 days. The conjugation efficiency was monitored by thin-layer chromatography (TLC). After dialysis and freeze-drying, the product ($DSPE\text{-}PEG_{2000}\text{-}ChA$) was obtained as white powder (18.2 mg, yield: 58%). The structure was confirmed by ^1H NMR. (600 MHz). ^1H NMR spectra of $DSPE\text{-}PEG_{2000}\text{-}NH_2$ and cholic acid standard sample were listed in supplementary Fig. S19 (a) and (c) respectively. As can be seen in supplementary Fig. S19 (b), the main proton peaks of

$DSPE\text{-}PEG_{2000}\text{-}NH_2$ and cholic acid were clearly observable and the carboxyl proton of cholic acid disappeared. Also, the ratio of the typical protons of cholic acid and the PEG protons of $DSPE\text{-}PEG_{2000}$ was about 3:180. Such results suggest cholic acid has been covalently linked to $DSPE\text{-}PEG_{2000}\text{-}NH_2$.

Preparation of liposomal $C^1X\text{-}OR^1$. 12.46 mg DPPC, 2.8 mg DSPC, 1.7 mg $DSPE\text{-}PEG_{2000}$-ChA (molar ratio: 85/10/5) were dissolved completely in 1 mL tetrahydrofuran, and then $C^1X\text{-}OR^1$ (20 mg, dissolved in 0.5 mL THF) was slowly added while stirring. The above mixture was injected slowly into in 10 mL phosphate buffer (pH 7.4, 10 mM). The organic solvent was removed by vacuum-rotary evaporation and then the mixture was dialyzed for 24 h. The suspension was centrifuged at 4 °C to collect the nanoparticles, liposomal $C^1X\text{-}OR^1$. After being washed for three times with water, the nanoparticles were resuspended in the phosphate buffer (or water for the following concentration determination) of designated volume (20 mL).

For determination of concentration of liposomal $C^1X\text{-}OR^1$ in water and $C^1X\text{-}OR^1$ loading, liposomal $C^1X\text{-}OR^1$ solution was freeze-dried and weighed. After that, freeze-dried liposomal $C^1X\text{-}OR^1$ was dissolved in 1 mL of dimethyl sulfoxide (DMSO). The loading amount of the $C^1X\text{-}OR^1$ was determined based on the absorbance intensity at 684 nm, according to the standard calibration curve experimentally obtained.

(1) Concentration of liposomal $C^1X\text{-}OR^1$ in water = (mass of nanoparticles) / (volume of solution)

(2) $C^1X\text{-}OR^1$ loading efficiency = (mass of loaded $C^1X\text{-}OR^1$) / (mass of nanoparticles) × 100%

The concentration of liposomal $C^1X\text{-}OR^1$ was 0.7 mg mL^{-1} and $C^1X\text{-}OR^1$ loading efficiency was determined as 42.7%.

Cell culture. Murine aneuploid fibrosarcoma cell line (L929), human hepatocarcinoma cell line (HepG2), human Ovarian Carcinoma Cell Lines (OVCAR3, SKOV3) were obtained from KeyGen Biology Co. Ltd (Nanjing, China). Human ovarian serous cystadenocarcinoma-derived cell line (SHIN3) was obtained from Shanghai Huiying biological technology Co. Ltd. All cell lines tested negative for mycoplasma contamination. No mycoplasma contamination was confirmed by the suppliers. L929, OVCAR3 and SHIN3 cells were cultured in complete medium containing RPMI1640 supplemented with 10% FBS (fetal bovine serum, GIBCO) and 1% penicillin and streptomycin (GIBCO) at 37 °C, 5% CO2. HepG2 cells were cultured in Dulbecco's modified eagle medium (DMEM) supplemented with 10% FBS and 1% penicillin and streptomycin at 37 °C under 5% of CO_2. SKOV3 cells was cultured in McCoy's 5A supplemented with 10% FBS and 1% penicillin and streptomycin at 37 °C under 5% of CO_2. When the cell density reached 70-80% of confluence, subculturing was considered complete. The medium was changed approximately every 1-2 days.

Body weight measurements. As for part of the evaluation of the probes' in vivo toxicity, body weights of mice were measured. For the body weight measurements, male BALB/c nude mice (n = 9 per group, 8-week old) were intravenously administered with 8.75 mg kg^{-1} liposomal C^1X-OR^1 (equivalent to 3.7 mg kg^{-1} of C_1X-OR_1) or with no injection. Female BALB/c nude mice (n = 9 per group, 10-week old) were intraperitoneally administered with C^2X-OR^2 (6.4 mg kg^{-1}, dissolved in PBS containing 5% DMSO) or with no injection. The body weight of these mice was recorded for 7 days.

Determination of probes' sensitivity in euthanized mice. The sensitivity, which refers to the

minimum amount of the absorbing agents (the activated probes) necessary in order to be detectable using MSOT, was determined according to a previously reported procedure [3,4] to evaluate performance of the probes in disease detection. The mice (n=3 for each activated probe) was euthanized and immediately used for sensitivity determination. The activated probe C^1X-OH (or C^2X-OH) were dissolved in DMSO and then diluted with TRIS (or PBS) solution under stirring. A capillary tube (diam. 0.8 mm) containing C^1X-OH (or C^2X-OH) was rectally inserted into the euthanized mouse for imaging. The capillary tube was filled with C^1X-OH (or C^2X-OH) at the peak absorption coefficients decreasing exponentially from 2 to 0.04 cm^{-1}. For C^1X-OH, imaging was performed at the following wavelengths: 680 nm, 684 nm, 700 nm, 715 nm, 730 nm, 760 nm, 800 nm, 850 nm. For C^2X-OH, imaging was performed at the following wavelengths: 680 nm, 690 nm, 703 nm, 715 nm, 730 nm, 760 nm, 800 nm, 850 nm. Signal denoising was performed by averaging 20 frames per wavelength and the images were reconstructed using the backprojection algorithm and then demixed using guided ICA method.

Western blot analysis. Liver tissues were harvested and rinsed in cold sterile PBS and then homogenized in ice-cold RIPA Lysis Buffer (1/10, w/v) of Keygen Biotech) with protease inhibitor cocktails (0.1 %) and PMSF (1 mM, Keygen Biotech). Homogenates were centrifuged at 12000 rpm for 15 min, and supernatants were assayed for protein content using a BCA Protein Assay Kit (Keygen Biotech) according to instructions provided. SDS-PAGE loading buffer was added into the protein supernatants (1/4, v/v, Keygen Biotech) and then the mixture were boiled for 10 min (100 ℃). After brief centrifugation, the protein samples were stored at -20 ℃ until further use. The protein samples were separated by electrophoresis on SDS-PAGE gels and transferred to PVDF membranes. The PVDF membranes were blocked with the Western Blocking Buffer (Keygen

Biotech) for 1 h at room temperature. After washed by PBST three times, the membranes were incubated with primary antibodies (anti-cleaved caspase-3 [catalog No.: A11888], P-JNK [catalog No.: AP0276], or GAPDH [catalog No.: AC002], ABclonal) at 1:1000 dilution under 4 ℃ overnight. After washed by TBST 5 times, the membranes were incubated with HRP-conjugated secondary antibodies ([catalog No.: AS028, AS003], ABclonal) at 1:5000 dilution for 1 h. After washed by TBST 5 times, the membranes were subject to chemiluminescence detection (ECL Kit, Keygen Biotech) by Tanon 5200 Imaging System.

TUNEL assays. In brief, livers were fixed in 10% buffered formalin and embedded in paraffin. Sections of 5 μm were affixed to slides and then deparaffinized and rehydrated. Endogenous peroxidase activity was quenched in 3% H_2O_2 in methanol for 15 min. The slides were incubated with terminal deoxynucleotidyl transferase solution at 37 ℃ for 1h. After washed in PBS three times for 5 min each, the slides were incubated with Streptavadin-HRP solution for another 30 min. The sections were colorized with fresh DAB solution and then washed in water to end the reaction. After dried overnight, the slides were counterstained with hematoxylin, coverslipped and observed under an optical microscope.

CD206 Immunohistochemistry analysis

In brief, for immunohistochemistry analysis, tumor adjacent tissue and control tissue slides were deparaffinized, rehydrated in PBS, and then endogenous peroxidase activity was quenched by immersion in 3% H_2O_2 in methanol for 10 min. A nonspecific blocking procedure making use of 5% bovine serum albumin was performed before application of primary antibodies. The antibody anti-CD206 ([catalog NO.: A8301], ABclonal) was used at a 1:50 dilution. The incubation was

performed at 4 ℃ overnight. Then an Elivision™ plus Polyer HRP (Mouse) IHC Kit was used to amplify the signal of antigen-antibody binding according to the manufacturer's instructions. The slides were colorized with fresh DAB solution and then washed in water to end the reaction. After dried overnight, the slides were counterstained with hematoxylin, coverslipped and observed under an optical microscope.

Supplementary References

1. Tzoumas, S. et al. Statistical Molecular Target Detection Framework for Multispectral Optoacoustic Tomography. *IEEE Trans. Med. Imaging.* 35, 2534-2545 (2016).
2. Tzoumas, S. et al. Unmixing Molecular Agents From Absorbing Tissue in Multispectral Optoacoustic Tomography. *IEEE Trans. Med. Imaging.* 33, 48-60 (2014).
3. Hama, Y. et al. D-galactose receptor-targeted *in vivo* spectral fluorescence imaging of peritoneal metastasis using galactosamin-conjugated serum albumin-rhodamine green. *J. Biomed. Opt.* **12**, 051501 (2007).
4. Kazuki, K. et al. Hypoxia-Sensitive Fluorescent Probes for in Vivo Real-Time Fluorescence Imaging of Acute Ischemia. *J. Am. Chem. Soc.* **132**, 15846-15848 (2010).
5. Qian, B. Z. & Pollard, J. W. Macrophage diversity enhances tumor progression and metastasis. *Cell*. 141: 39-51(2010).
6. Yin, M. et al. Tumor-associated macrophages drive spheroid formation during early transcoelomic metastasis of ovarian cancer, *J. Clin. Invest.* **126**, 4157–4173(2016).
